# Altered Driving Structures and Controllability Patterns of Brain Effective Networks in Mild Cognitive Impairment and Alzheimer’s Disease

**DOI:** 10.3390/brainsci16080820

**Published:** 2026-07-31

**Authors:** Jiayue Xue, Xinyi Yan, Jing Wei, Min Gao, Ke Liang

**Affiliations:** 1 School of Information, Shanxi University of Finance and Economics, Taiyuan 030006, China; 2 School of Statistics, Shanxi University of Finance and Economics, Taiyuan 030006, China

**Keywords:** Alzheimer’s disease, mild cognitive impairment, brain effective network, driving structure, controllability index

## Abstract

**Highlights:**

**What are the main findings?**

- Diagnostic-group differences in threshold-derived driving structures were observed in structurally constrained directed brain effective networks, with default-mode regions acting as common drivers and additional sensorimotor drivers in MCI and frontoparietal drivers in AD.
- Controllability patterns varied by analytic scale: whole-brain, default-mode, and frontoparietal indices showed a nonmonotonic NC–MCI–AD pattern, whereas hub-level controllability showed the opposite pattern; regional node controllability was associated with cognitive scores.

**What are the implications of the main findings?**

- These findings suggest that MCI and AD are associated with changes in the brain’s network control architecture, providing insight into altered directed information propagation during cognitive decline.
- The controllability patterns of multimodal brain effective networks may provide a framework for characterizing network-level differences across the AD clinical spectrum and for motivating future longitudinal validation.

**Abstract:**

**Background:** Alzheimer’s disease (AD) and mild cognitive impairment (MCI) are associated with abnormalities in brain networks. However, differences in the control architecture of directed brain effective networks among normal controls (NC), patients with MCI, and patients with AD remain insufficiently characterized. This study investigated diagnostic-group differences in driving structures and controllability patterns in structurally constrained brain effective networks. **Methods:** Multimodal neuroimaging data, including diffusion MRI and resting-state functional MRI, were used to construct brain effective networks in the NC, MCI, and AD groups. Directed interregional interactions were estimated using multivariate autoregressive modeling under structural connectivity constraints. A structural controllability framework based on maximum matching was then applied to identify group-level driving nodes and driving edges. Controllability index was compared across groups separately at the whole-brain, resting-state network (RSN), and regional levels. **Results:** Group-level driving structures differed among the NC, MCI, and AD groups. Driving nodes were mainly distributed within the default mode network, with additional somatosensory and motor network drivers in MCI and a frontoparietal network driver in AD. The whole-brain controllability index decreased from NC to MCI and increased from MCI to AD. Similar nonmonotonic patterns were observed in the default mode and frontoparietal networks, whereas the somatosensory and motor and visual networks showed different group patterns. Regional node controllability was negatively associated with indegree and positively associated with outdegree, and driving nodes were more likely to correspond to outdegree hubs. Hub-level controllability showed a pattern opposite to that of the whole brain. Several regional indices were associated with cognitive scores, although these pooled cross-sectional associations may partly reflect diagnostic-group separation. **Conclusions:** MCI and AD were associated with differences in driving structures and controllability patterns in directed brain effective networks. These findings provide preliminary network-level observations on altered directed information propagation across the AD clinical spectrum and require validation in larger, independent, and longitudinal cohorts.

## 1. Introduction

Alzheimer’s disease (AD) is a progressive neurodegenerative disorder characterized by memory loss and cognitive decline, whereas mild cognitive impairment (MCI) is commonly regarded as a transitional clinical stage between normal cognition (NC) and AD [1,2]. Neuroimaging studies have shown that cognitive impairment in MCI and AD is associated with abnormalities in both structural connectivity (SC) and functional connectivity (FC) of brain networks [3,4,5,6,7]. However, SC and FC provide limited information about the directionality of interregional interactions. Effective connectivity (EC), by contrast, estimates directed statistical dependencies among brain regions and may provide complementary information about abnormal interregional communication during cognitive decline [8,9]. Integrating SC and EC may provide a more biologically constrained representation of directed brain networks, because anatomical pathways can support and constrain functional interactions between brain regions.

Beyond describing network connectivity, network controllability provides a theoretical framework for examining how network topology supports transitions among system states [10,11,12]. In this framework, the network topology helps identify brain regions and connections that may play key roles in driving overall brain activity. Previous studies have applied controllability analyses to structural or functional brain networks and identified regions that may contribute to cognitive state transitions [12,13,14]. However, SC-based analyses do not directly estimate functional directionality, whereas FC-based analyses are generally undirected. A structurally constrained directed effective network may therefore provide a complementary framework for characterizing diagnostic-group differences in brain network control. It remains necessary to determine whether the organization of driving nodes and driving edges differs among NC, MCI, and AD groups.

Structural controllability theory provides an approach for identifying a minimum set of driver nodes required to control a directed complex network [15,16]. Through the minimum-input theorem and maximum-matching algorithms, this framework identifies network elements that are topologically important for controllability [17,18]. Applying this approach to structurally constrained brain effective networks may help characterize group differences in driving structures and provide a network-level perspective on altered directed information propagation in MCI and AD.

In the present study, we constructed structurally constrained brain effective networks using multimodal neuroimaging data. Directed interregional interactions were estimated using multivariate autoregressive modeling under SC constraints. We then applied a structural controllability framework to identify group-level driving nodes and driving edges in NC, MCI, and AD groups. Finally, we defined a driver-edge-based controllability index and compared it across groups at the whole-brain, resting-state-network, and regional levels. Compared with controllability analyses based solely on SC or FC, this framework incorporates both anatomical constraints and directed statistical interactions. We aimed to characterize diagnostic-group differences in network control architecture and to generate hypotheses regarding altered directed information propagation across the AD clinical spectrum.

## 2. Materials and Methods

### 2.1. Participants

All neuroimaging data used in this study were obtained from two publicly available datasets: the Human Connectome Project (HCP) Retest dataset (http://www.humanconnectome.org/ (accessed on 29 July 2026)) and the Alzheimer’s Disease Neuroimaging Initiative (ADNI) database (http://adni.loni.usc.edu (accessed on 29 July 2026)). The imaging data included T1-weighted structural MRI, diffusion tensor imaging (DTI), and resting-state functional MRI (rs-fMRI).

The HCP Retest dataset included 45 healthy participants who underwent repeated MRI acquisitions. These data were used to evaluate test–retest reproducibility and to examine the sensitivity of SC–EC integration to penalty-parameter choices.

The ADNI dataset included 77 participants, consisting of 28 normal controls (NC; mean age = 73.18 ± 7.96 years; 15 females), 33 patients with mild cognitive impairment (MCI; mean age = 76.53 ± 9.48 years; 16 females), and 16 patients with Alzheimer’s disease (AD; mean age = 74.55 ± 9.15 years; 8 females). All participants were right-handed and had no history of other neurological or psychiatric disorders. Clinical cognitive assessments included the Mini-Mental State Examination (MMSE), Clinical Dementia Rating (CDR), and Functional Activities Questionnaire (FAQ). Demographic and clinical characteristics are summarized in Table 1. No significant differences were observed among the groups in age (F(2, 74) = 1.116, *p* = 0.333, *η*^2^ = 0.029) or sex distribution (χ^2^(2) = 0.160, *p* = 0.923, Cramér’s V = 0.046). CDR, FAQ, and MMSE scores differed among the three groups (all *p* < 0.001).

### 2.2. Data Acquisition and Preprocessing

For the HCP Retest dataset, MRI data were acquired using a customized Siemens Connectome Skyra 3T scanner (Siemens Healthcare, Erlangen, Germany) with a 32-channel head coil. Each participant completed two rs-fMRI scanning sessions on separate days. During scanning, participants were instructed to relax, keep their eyes closed, and remain awake. Each rs-fMRI session included two resting-state runs acquired with 2 mm isotropic resolution, repetition time (TR) = 720 ms, field of view (FOV) = 210 × 180 mm^2^, flip angle = 52°, and 72 slices. T1-weighted MRI data were acquired with 0.7 mm isotropic resolution, TR = 2400 ms, echo time (TE) = 2.14 ms, FOV = 224 × 224 mm^2^, flip angle = 8°, and 320 slices. DTI data were acquired with TR = 5520 ms, TE = 89.5 ms, FOV = 210 × 180 mm^2^, flip angle = 78°, 111 slices, and 1.25 mm isotropic voxel size.

For the ADNI dataset, rs-fMRI data were acquired using Siemens Trio 3T scanners. The rs-fMRI acquisition parameters were as follows: 48 slices, slice thickness = 3.3 mm, TR = 3000 ms, TE = 30 ms, FOV = 192 × 192 mm^2^, flip angle = 80°, matrix = 64 × 64, and voxel size = 3 × 3 × 3 mm^3^. T1-weighted MRI data were acquired with 176 slices, TR = 2300 ms, TE = 2.98 ms, FOV = 240 × 256 mm^2^, flip angle = 9°, matrix = 240 × 256, and voxel size = 1 × 1 × 1 mm^3^. DTI data were acquired with 31 slices, slice thickness = 2.0 mm, TR = 12,400 ms, TE = 95 ms, flip angle = 90°, and 54 diffusion directions.

Diffusion-weighted MRI data were acquired using a single-shell protocol comprising non-diffusion-weighted images at b = 0 s/mm^2^ and diffusion-weighted images at a single non-zero b-value of 1000 s/mm^2^. Therefore, the acquisition did not contain multiple non-zero diffusion-weighting shells.

T1-weighted MRI data were preprocessed using FreeSurfer (version 7.2.0, Athinoula A. Martinos Center for Biomedical Imaging, Massachusetts General Hospital, Boston, MA, USA) [19]. The preprocessing procedures included motion correction, skull stripping, tissue segmentation, spatial normalization, cortical reconstruction, and anatomical parcellation.

DTI data were preprocessed using FSL (version 6.0, FMRIB Centre, University of Oxford, Oxford, UK) [20], FreeSurfer, and MRtrix3 (version 3.0, Brain Research Institute, Monash University, Melbourne, Australia). The preprocessing procedures included correction for eddy-current distortions and head motion, diffusion tensor estimation, and reconstruction of white matter pathways. High-resolution T1-weighted images were used to generate anatomical boundary masks and (Region of Interest) ROI templates. White matter fiber tracking was then performed to estimate structural connectivity between brain regions. The weight of each structural connection was defined according to the number of reconstructed fibers between each pair of ROIs and was normalized by cortical surface area.

Rs-fMRI data were preprocessed using the Data Processing Assistant for Resting-State fMRI toolbox (DPARSF, version 5.0, State Key Laboratory of Cognitive Neuroscience and Learning, Beijing Normal University, Beijing, China) and SPM (version 12, Wellcome Centre for Human Neuroimaging, London, UK) [21]. The first 10 volumes were removed to allow for signal stabilization. Slice timing was applied to eliminate the impact of different acquisition times on the data. Realignment to compensate for head movement was applied using a 6-parameter rigid-body spatial transformation. Head motion was quantified using mean framewise displacement (mean FD) derived from the realignment parameters, and mean FD did not differ significantly among the NC, MCI, and AD groups. All images were normalized to Montreal Neurological Institute space, and resampled to 3 × 3 × 3 mm^3^. Linear trends were removed, and spatial smoothing was performed using a Gaussian kernel with a full width at half maximum of 6 mm. Temporal band-pass filtering was applied within 0.01–0.08 Hz. Nuisance covariates, including the Friston-24 parameters, global mean signal, white matter signal, and cerebrospinal fluid signal, were regressed out before subsequent analyses [22].

### 2.3. Construction of Structurally Constrained Brain Effective Network

A network comprises nodes and edges connecting the nodes. In order to analyze the directed brain functional network more accurately and reliably, the Desikan Killiany atlas implemented in FreeSurfer was used to divide the cerebral cortex into 68 gyral-based cortical regions of interest, with 34 brain regions symmetrically distributed in the left and right hemispheres [23]. The specific names and resting-state network (RSN) information of all brain regions are shown in the Supplementary Materials (Table S1).

Using a multivariate autoregressive (MVAR) model, Granger causality analysis (GCA) was applied to the ROI time series to estimate directed effective connectivity between each ROI and all other ROIs. For two time series *X*(*t*) and *Y*(*t*), GCA can be formulated as follows:

(1) $$Y_{t}=\sum_{i=1}^{p}A_{i}X_{\left(t-i\right)}+\sum_{i=1}^{p}B_{i}Y_{\left(t-i\right)}+CZ_{t}+\varepsilon_{t}$$

(2) $$X_{t}=\sum_{i=1}^{p}A_{i}^{\prime }Y_{\left(t-i\right)}+\sum_{i=1}^{p}B_{i}^{\prime }X_{\left(t-i\right)}+C^{\prime }Z_{t}+\varepsilon_{t}^{\prime }$$

where *p* is the model order, i.e., the maximum number of lagged samples, $A_{i}$ (Formula (1)) and $A_{i}^{\prime }$ (Formula (2)) are the signed-path coefficients, $B_{i}$ (Formula (1)) and $B_{i}^{\prime }$ (Formula (2)) are autoregression coefficients, $\varepsilon_{t}$ (Formula (1)) and $\varepsilon_{t}^{\prime }$ (Formula (2)) are residuals, and $Z_{t}$ represents the covariates (e.g., head motion, global trend). If ε is reduced by the inclusion of X, then X is considered to be the causality of Y. If $\varepsilon^{\prime }$ is reduced by the inclusion of Y, then Y is considered to be the causality of X. The magnitude of the interaction could be measured by the log ratio of the prediction error variances for the restricted (*R*) and unrestricted (*U*) models [24]:

(3) $$F_{X\to Y}=ln\frac{\mathrm{var}(\varepsilon_{R})}{\mathrm{var}(\varepsilon_{U})}$$

(4) $$F_{Y\to X}=\ln\frac{\mathrm{var}(\varepsilon_{R}^{\prime })}{\mathrm{var}(\varepsilon_{U}^{\prime })}$$

where $\varepsilon_{R}$ and $\varepsilon_{R}^{\prime }$ are derived from the model omitting the $A_{i}$ and $A_{i}^{\prime }$ (for all *i*) coefficients. $\varepsilon_{U}$ and $\varepsilon_{U}^{\prime }$ are derived from the full model. GCA is considered statistically significant if the signed-path coefficients $A_{i}$ and $A_{i}^{\prime }$ are significantly different from zero, which could be identified by F-test (Bonferroni correction, threshold is $P_{\mathrm{nom}}/n(n-1)$, $P_{\mathrm{nom}}$ = 0.05) [25].

Following statistical testing, nonsignificant coefficients were set to zero. Statistically significant negative signed-path coefficients were also set to zero; thus, only statistically significant positive coefficients were retained to construct the directed effective connectivity matrix. This positive-edge restriction yielded a nonnegative matrix compatible with the subsequent fractional-power SC–EC fusion. The treatment of negative edges represents a methodological choice in graph construction, and alternative approaches that explicitly retain and analyze signed edges may yield different network topologies [26,27].

For the model order *p*, too small *p* can lead to a poor representation of the data, whereas too large can lead to problems of model estimation. Therefore, we used Akaike Information Criterion (AIC) [28] and Bayesian Information Criterion (BIC) [29] to select the optimal model order *p*. For *n* variables:

(5) $$\mathrm{AIC}\left(p\right)=\ln\left(\det\left(\Sigma \right)\right)+\frac{2pn^{2}}{T}$$

(6) $$\mathrm{BIC}\left(p\right)=\ln\left(\det\left(\Sigma \right)\right)+\frac{\ln(T)pn^{2}}{T}$$

where Σ is the noise covariance matrix of the restricted model, T is the time series length. The results showed that, whether AIC or BIC was used, the error noise was the smallest shown (Figure S1) when the model order *p* was selected as 1.

Based on DTI data, this chapter first calculates Fractional Anisotropy (FA) for each voxel, and uses affine transformation to register FA images into corresponding T1-weighted images. Diffusion tensors were estimated after correction for eddy-current distortions and head motion. Deterministic whole-brain tractography was subsequently performed using the Fiber Assignment by Continuous Tracking algorithm. Streamlines were assigned to pairs of cortical regions according to their reconstructed endpoints, and a subject-level tractography-derived structural connectivity matrix was constructed. The weight of each edge was defined as the number of reconstructed streamlines connecting the corresponding pair of regions and was normalized according to cortical surface area. In the present framework, this matrix was used as an anatomical prior for constraining the MVAR model. Reconstructed streamline counts were not interpreted as direct measurements of axonal number, axonal density, neurite density, or absolute biological connection strength.

According to the above MVAR model definition, an MVAR model can be viewed as a random process, $Y_{t}$ with r variables linearly dependent on their own previous values and random terms, can be defined as:

(7) $$Y_{t}=\sum_{i=1}^{p}A_{i}Y_{\left(t-i\right)}+CZ_{t}+\varepsilon_{t}$$

where p is the order of the MVAR model, $A_{i}$ is the signed path coefficient matrix between the r variables, $\varepsilon_{t}$ are the residuals, and $Z_{t}$ represents the covariates (e.g., head motion and global trend). The weight matrix $A_{i}$ describes the lagged linear relationships between r time series ${\left[y_{1},\;y_{2},\ldots ,\;y_{r}\right]}^{T}$. Therefore, they can be understood as FC matrices with different time lags, with r representing time lags.

In the present study, the model order was set to *p* = 1, indicating that the current state of each node was estimated from the immediately preceding time point. Multivariate autoregressive models have been widely applied to characterize directed interactions in fMRI time series, providing a framework for estimating effective connectivity from temporal dependencies among brain regions [30]. Considering the relatively short resting-state fMRI time series and the high dimensionality of whole-brain networks, a first-order model was adopted to balance model complexity and parameter estimation stability, as higher-order models require estimation of additional parameters and may increase the risk of unstable estimation in high-dimensional settings [31].

**Figure 1:**
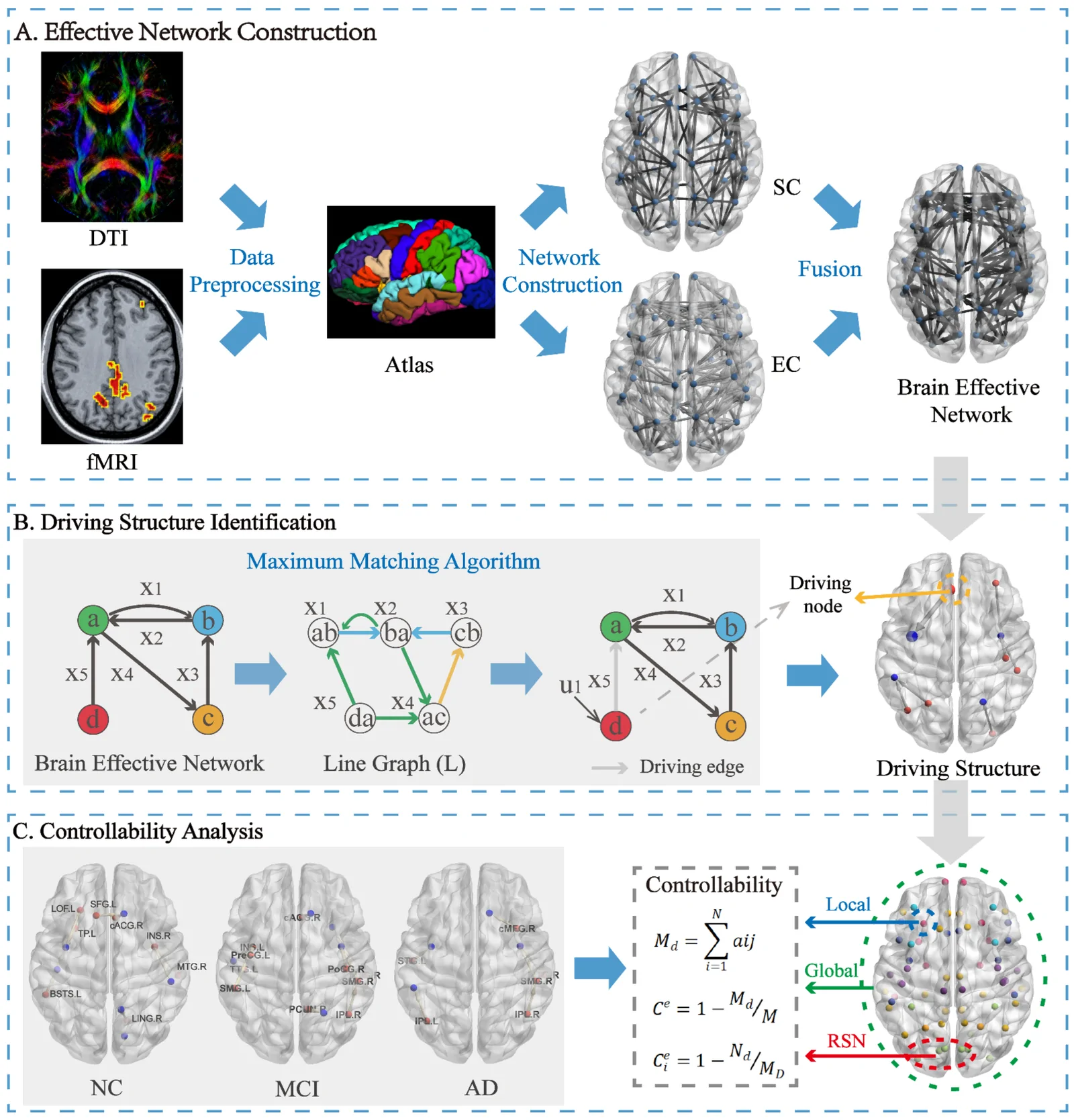
Analytical workflow for constructing structurally constrained brain effective networks and evaluating driving structures and controllability. (**A**) Brain effective network construction: diffusion MRI and rs-fMRI preprocessing, cortical parcellation using the Desikan-Killiany atlas, construction of SC and directed EC matrices, and SC–EC integration. (**B**) Driving structure identification using maximum matching. (**C**) Calculation of controllability index at the whole-brain, resting-state network (RSN), and regional levels.

This study aims to strengthen the direct relationship of EC by fusing the SC information, so that the direct physical connectivity of brain regions can assist in describing the functional information interaction of the brain (Figure 1A). To reduce anatomically unsupported directed interactions, the tractography-derived structural connectivity matrix was incorporated as a prior constraint in the MVAR model. The resulting network should therefore be interpreted as a structurally informed estimate of directed interregional interactions rather than as a direct measurement of causal communication or complete anatomical connectivity.

(8) $$\hat{A_{i}}=A_{i}^{\alpha }⊙B^{\beta }$$

Here, ⨀ represents the product of matrix elements, $A_{i}$ represents the directed effective connectivity matrix, *B* represents the index matrix obtained by the SC matrix, and α and β are penalty parameters. To ensure the rationality of the SC matrix, B contains both direct and indirect SC information. Specifically, B can be defined as:

(9) $$B=B_{d}+B_{\mathrm{ind}}$$

Here, $B_{d}$ denotes the direct SC matrix, $B_{\mathrm{ind}}$ denotes the indirect SC matrix, and the original SC matrix is $B_{d}$ The direct matrix was binarized to obtain $B_{b}$ For each ordered region pair (*x*,*y*), $d_{xy}^{B_{b}}$ and $d_{xy}^{B_{d}}$ denote the shortest path lengths in the binary and weighted structural networks, respectively. The structural modulation factor $b_{xy}$ was then defined as follows:

(10) $$b_{xy}=\frac{d_{xy}^{B_{d}}}{d_{xy}^{B_{b}}}$$

The purpose of this coefficient is to characterize the relative structural accessibility between brain regions. Unlike direct weighting approaches that mainly consider the strength of anatomical connections, shortest-path-based measures incorporate information from both direct and indirect anatomical routes, thereby providing a network-level constraint on information transmission [32,33]. Therefore, the proposed framework retains the directionality of effective interactions while weighting their strength according to structural accessibility.

In order to evaluate the fusion effect of SC, penalty parameters α and β value is set between 0 and 1, with an interval of 0.1. Based on the healthy participants who have undergone two repeated measurements in the HCP database, this study conducted the Intraclass Correlation Coefficient (ICC) analysis on the brain effective networks of the two groups. The ICC values were calculated according to the following equation:

(11) $$\mathrm{ICC}\;=\;\frac{{\mathrm{MS}}_{\lambda }-{\mathrm{MS}}_{\varepsilon }}{{\mathrm{MS}}_{\lambda }+(k-1){\mathrm{MS}}_{\varepsilon }+\frac{k}{n}({\mathrm{MS}}_{\pi }-{\mathrm{MS}}_{\varepsilon })}$$

${\mathrm{MS}}_{\lambda }$ is the between-subject mean square, ${\mathrm{MS}}_{\varepsilon }$ is the residual mean square, ${\mathrm{MS}}_{\pi }$ is the between-scan mean square, *n* is the number of subjects, and *k* is the number of repeated observations per subject. Here, ICC values close to 1 indicate perfect agreement between two scans, while ICC values close to 0 (or negative) indicate poor or no agreement. In order to balance the proportion of SC and EC such that SC did not affect the true direct relationship within the EC, the final selection for this study were α = 0.8 and β = 0.1. Penalty parameters *α* and *β* were initially examined over the 0.1–1.0 grid. A complete one-step local sensitivity window was then evaluated around the adopted *α* = 0.8, *β* = 0.1 setting. Because *β* = 0.1 was the lower boundary of the 0.1-spaced grid, the local window comprised *α* = {0.7, 0.8, 0.9} and *β* = {0.1, 0.2}, yielding six settings. HCP reproducibility was evaluated using edgewise test–retest ICC. In the ADNI normal-control subgroup, each resting-state time series was divided into two contiguous, nonoverlapping segments of equal length, and within-scan split-half agreement was quantified using Spearman correlation (Figure S2). Because the ADNI segments originated from the same acquisition and shared the same SC matrix, this analysis was interpreted as a sensitivity analysis rather than an independent test–retest validation. EC rank retention and SC alignment were additionally quantified for each setting. The *α* = 0.8, *β* = 0.1 setting was retained solely as the adopted reference setting for comparability. Detailed results for the six-setting local sensitivity window are provided in Table S2.

### 2.4. Identification of Driving Nodes and Driving Edges

Based on the derived brain effective network, its control network dynamics are represented as follows:

(12) $$y_{i}^{+}\left(t\right)=M_{i}y_{i}^{-}\left(t\right)-\tau_{i}⊗y_{i}^{+}\left(t\right)+\sigma_{i}u_{i}\left(t\right)$$

Here, $y_{i}^{-}$ and $y_{i}^{+}$ are vectors composed of the values $x_{j}$ of the incoming and outgoing edges at vertex i, respectively. Let $M_{i}$ denote a matrix whose number of rows equals the out-degree of vertex i, and its number of columns equals the in-degree of that vertex. $\tau_{i}$ is the damping term vector corresponding to the edge in $y_{i}^{+}\left(t\right)$; if vertex i is a so-called driving node, $\sigma_{i}$ equals 1; otherwise, it equals 0. ⨂ the Hadamard product of two vectors of equal magnitude.

Let $x\;=\;[x_{j}]$ denote the state vector of the control process; then each edge in the network can correspond to a state variable. For any edge *j* that originates from a vertex *r* and terminates at vertex s, the derivative of its state depends only on its own state and the states of edges whose source is r. Here, $\Gamma_{j}^{-}$ denotes the set of edges whose source is r, and the dynamical equation can be simplified as:

(13) $$\dot{x_{j}}=\sum_{k\in \Gamma_{j}^{-}}w_{kj}x_{k}-\tau_{j}x_{j}+\sigma_{s}u_{j}$$

where $w_{kj}$ is the element in the mixing matrix $M_{r}$ of vertex r corresponding to edge k (as an incoming edge) and edge j (as an outgoing edge), $\tau_{j}$ is the damping term associated with edge j, and $u_{j}$ is the input signal value affecting the state variable of edge j. For all $k\notin \;\Gamma_{j}^{-}$, defining $w_{kj}$ = 0, we obtain:

(14) $$\dot{x}=\left(W-T\right)x+Hu$$

The unknown variables here are as follows: *W* = [$w_{kj}$] is a matrix in which $w_{kj}$ can be nonzero if and only if the source of edge k is the target of edge j. T is a diagonal matrix whose diagonal entries are the damping terms for each edge. H is a diagonal matrix in which the *j-th* diagonal element is s if vertex *s* is the target of edge *j*.

Formula (14) essentially describes a simple linear time−invariant dynamical system of the form:$\;\dot{x}=Ax+Bu,$ where *A* = *W* − *T*, *B* = *H*. Under this formulation, *W* is the transpose of the adjacency matrix of the directed line graph *L*(*G*) of the generated brain effective network. Therefore, the nodes of *L*(*G*) correspond to the edges of the generated brain effective network, and each edge of *L*(*G*) represents two consecutively connected directed edges in *G*. An example of the generated brain effective network is shown in Figure 1B, and the corresponding line graph of the generated brain effective network is shown in Figure 1B.

Applying the maximum-matching algorithm to the directed line graph yielded a set of control paths and unmatched line-graph nodes, which corresponded to driving edges in the original brain effective network (Figure 1B). The source nodes of these driving edges were defined as driving nodes.

### 2.5. Group-Level and Subject-Specific Driving Structures

**Figure 2:**
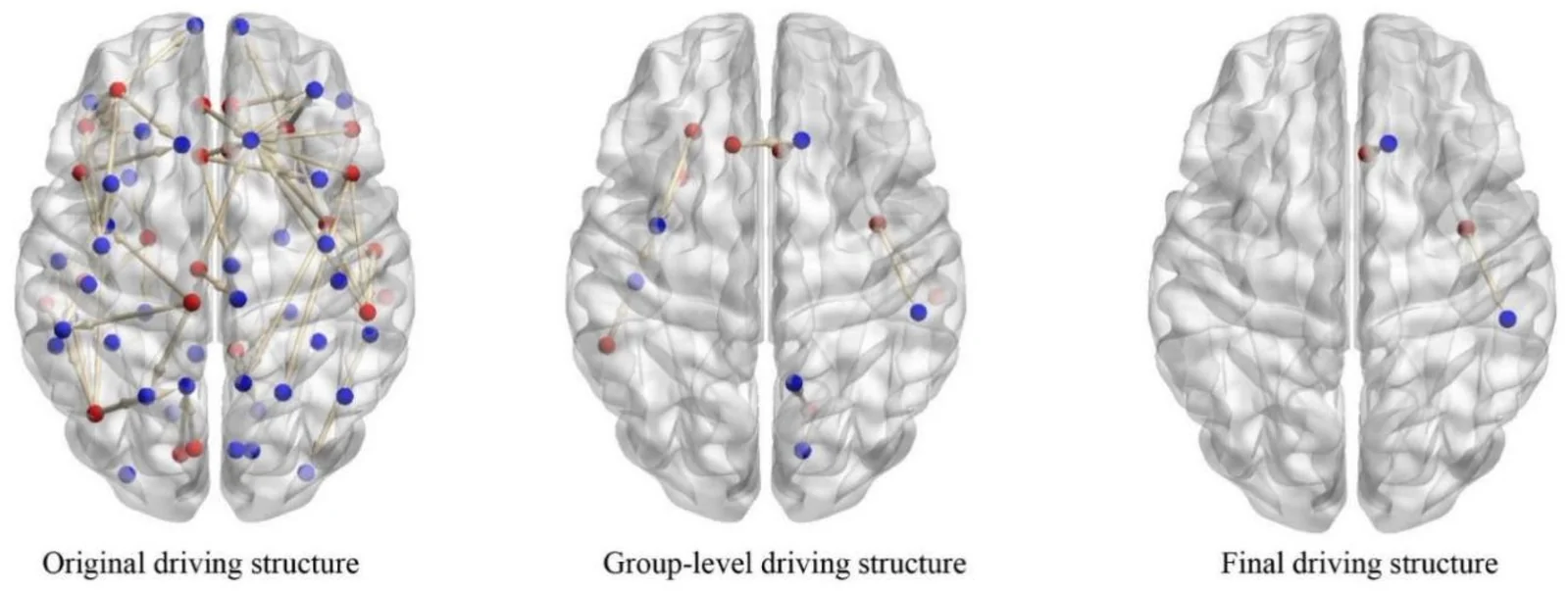
Construction of subject-level and group-level driving structures. **Left**: an initial driving structure for an NC participant. Red nodes denote driving nodes, outgoing edges from these nodes denote driving edges, and blue nodes denote controlled nodes. **Middle**: the NC group-level driving structure obtained from recurrent driving edges across participants. **Right**: the final subject-specific driving structure obtained by intersecting the participant-level and corresponding group-level driving-edge sets.

For each participant, the maximum-matching algorithm was applied to the directed brain effective network to identify an initial subject-level driving structure consisting of driving edges, their source driving nodes, and the associated controlled nodes (Figure 2, left).

To construct the group-level driving structure, the occurrence of each driving edge was counted across the initial driving structures of all participants within each diagnostic group. A driving edge was retained when its occurrence proportion reached the specified group-level occurrence threshold. The source nodes of the retained driving edges were defined as group-level driving nodes, and the corresponding connected nodes were included as group-level controlled nodes (Figure 2, middle).

For each participant, the final subject-specific driving structure was obtained by retaining the driving edges that were present in both the participant’s initial driving structure and the group-level driving structure of the corresponding diagnostic group. The source nodes and connected nodes of these retained edges formed the final subject-specific driving structure (Figure 2, right). Unless otherwise specified, the subject-specific driving structure used in the subsequent analyses refers to this final structure.

Because the number of participants contributing to an edge is an integer, nominal percentage thresholds correspond to minimum participant counts. Consequently, the retained driving structure changes in a stepwise rather than continuous manner, and adjacent percentage thresholds may produce identical edge and node sets. Threshold dependence was further examined at occurrence thresholds of 10, 16, 22, 28, and 35%. A 22% occurrence threshold was used for the main analysis. For the AD group, these thresholds corresponded to minimum occurrence counts of 2, 3, 4, 5, and 6 participants, respectively.

### 2.6. Controllability Index of Brain Effective Networks

Based on the data-driven group-level structural framework, the controllability index of the brain effective network is expressed as follows:

(15) $$M_{d}=\sum_{i=1}^{N}aij,j=1,2,\ldots ,N$$

(16) Ce=1−MdM

For the driver substructure of each subject, the number of driver edges $M_{d}$ in the driver substructure can be obtained using Formula (15), where *N* is the number of nodes in the brain effective network, aij is the binary effective connection from node *i* to node *j* in the brain effective network corresponding to the driver substructure, and *M* is the number of edges in the brain effective network. Using Formula (16), the controllability index $C^{e}$ of each subject’s brain effective network can be calculated. Within this driver-edge-based formulation, a higher index indicates a smaller proportion of driving edges relative to the denominator M. It should not be interpreted as lower control energy, greater clinical modulability, or greater ease of external stimulation.

In addition to the whole-network index, a regional node controllability index was calculated for each node as follows:

(17) Cie=1−NdMD,i=1,2,…,N

For each node on all matched paths in the network, the shortest number of nodes $N_{d}$ between this node and the driver node along the path can be calculated. If a node appears in different matched paths, the $N_{d}$ values of the node in different matched paths are calculated separately, and then the minimum value among them is selected as the final result. $M_{D}$ is the number of driver edges in the driver substructure. For a brain effective network with *N* nodes, the C values of the *N* nodes can be obtained.

### 2.7. Identification of Hub Nodes

Indegree and outdegree values were standardized using z scores, and nodes were classified separately according to incoming and outgoing centrality.

(18) $$Z_{i}^{in}=\frac{O_{i}^{in}-\langle o^{in}\rangle }{\sigma^{in}}$$

(19) $$Z_{i}^{out}=\frac{O_{i}^{out}-\langle o^{out}\rangle }{\sigma^{out}}$$

where 〈o〉 is equal to the mean overlapping indegree and outdegree of all nodes, and σ represents the corresponding standard deviation. Since the Z-score ranges calculated from the indegree (*Z-score-i*) and outdegree (*Z-score-o*) were inconsistent, the study used the last 1/3 *Z-score-i* and *Z-score-o* ranges of NC, MCI and AD as the hub node standard and the first 2/3 *Z-score-i* and *Z-score-o* ranges of NC, MCI and AD as the nonhub node standard based on the existing division standards [34].

The 1/3 threshold was adopted following established practices in brain network hub identification, where hubs are commonly defined as nodes with degree or centrality values exceeding a specified percentile of the population distribution [11,35]. This approach provides a reproducible and data-driven criterion for distinguishing relatively high-centrality regions and has been widely used in prior studies of brain network organization. We acknowledge that alternative thresholds (e.g., top 10% or top 20%) or classification strategies may yield different hub assignments; however, the percentile-based method employed here offers a straightforward and commonly accepted operational definition for identifying hubs in the context of group-level network comparisons.

Finally, all nodes are classified into four types: hub nodes for information reception (*i-hub*), hub nodes for information transmission (*o-hub*), non-hub nodes for information reception (*i-nonhub*), and non-hub nodes for information transmission (*o-nonhub*).

### 2.8. Statistical Analysis

Statistical analyses were performed using SPSS (version 26.0, IBM Corp., Armonk, NY, USA). Continuous variables are presented as mean ± standard deviation (SD). Differences in sex distribution among diagnostic groups were examined using the Pearson chi-square test. Differences in age and clinical scores were evaluated using one-way ANOVA followed by Bonferroni-corrected post hoc comparisons. Effect sizes were reported as Cramér’s V for the chi-square test and eta-squared (*η*^2^) for one-way ANOVA.

Group differences in global, hemispheric, RSN, regional, and hub-level indices were evaluated using one-way ANOVA. Omnibus effects were reported using F statistics, degrees of freedom, *p* values, and partial eta-squared (*ηp*^2^). For the 68 regional outcomes, the Benjamini–Hochberg (BH) procedure was applied across the 68 omnibus *p* values. Post hoc pairwise comparisons were conducted for regions meeting the regional BH-FDR (false discovery rate) criterion. Within each selected region, the three pairwise contrasts (NC–MCI, NC–AD, and MCI–AD) were corrected using the Bonferroni procedure. Pairwise results were reported as mean differences with confidence intervals, adjusted *p* values, and signed Hedges’ g values.

Whole-brain and regional associations with clinical scores were evaluated using hierarchical second-order polynomial regression. Age and sex were entered in Block 1. The index was mean-centered, and its centered linear and squared terms were entered jointly in Block 2. Incremental contribution was summarized using ΔR^2^ and the F-change test. The three whole-brain models and 204 regional models (68 regions × 3 scores) were treated as distinct multiplicity families and corrected separately using BH-FDR. Because participants were pooled across diagnoses, these models were interpreted as exploratory cross-sectional associations; the block-level test was not used to infer a specific curvature direction.

Additional sensitivity, reproducibility, and uncertainty analyses were performed for the fusion parameters, occurrence thresholds, and whole-brain *C*.

For the occurrence-threshold sensitivity analysis, the group-level and final subject-specific driving structures were reconstructed at thresholds of 10, 16, 22, 28, and 35%. Within the AD group, these thresholds corresponded to minimum occurrence counts of 2, 3, 4, 5, and 6 participants, respectively. At each threshold, the number of retained group-level driving edges was recorded, and structural similarity was quantified using Jaccard and Dice coefficients relative to the structure obtained at 22%.

Because the thresholded edge sets were nested and the 22% structure was compared with itself, the similarity coefficients were treated as descriptive measures of structural change. Sampling variability was assessed using 1000 within-group subject-level bootstrap resamples. Node prevalence across the five threshold settings was also recorded. Whole-brain *C* and the corresponding group comparisons were recalculated at each threshold.

## 3. Results

Brain effective networks were constructed for the NC, MCI, and AD groups using structurally constrained directed interregional interactions. Structural controllability analysis was then applied to identify driving structures, including driving nodes and driving edges. Controllability index was examined at the whole-brain, RSN, and regional levels. The RSNs examined in this study included the somatosensory and motor network (SMN), visual network (VN), default mode network (DMN), frontoparietal network (FPN), ventral attention network (VAN), and limbic network (LIM) (Supplementary Materials Table S1).

### 3.1. Diagnostic-Group Differences in Driving Structures

**Figure 3:**
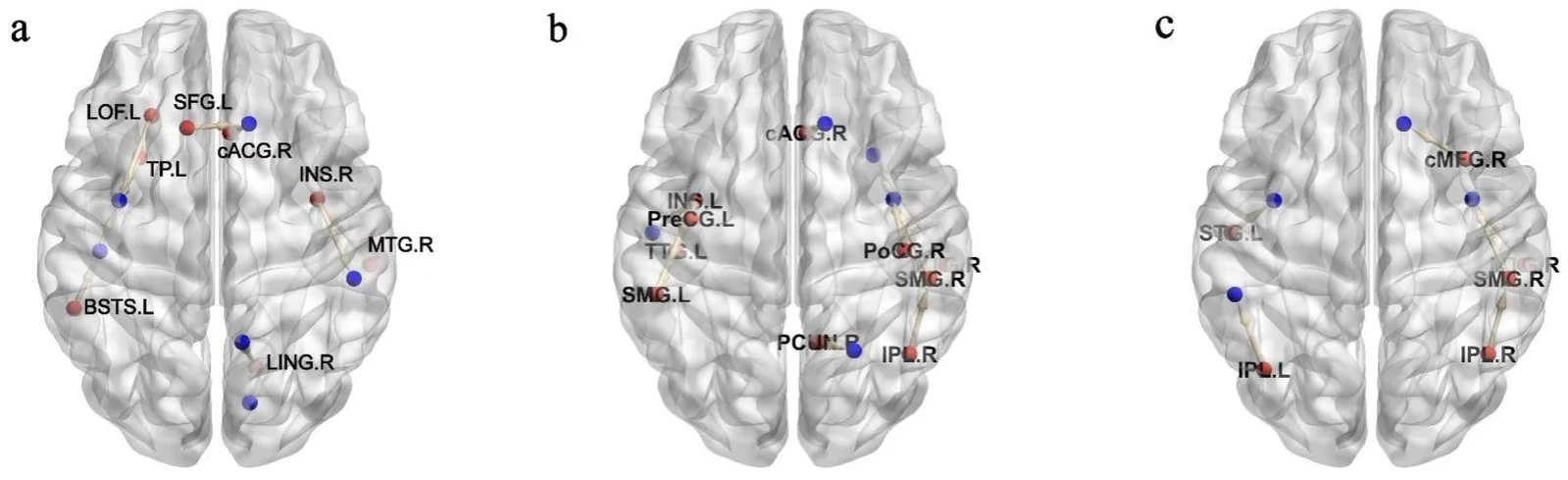
Group-level driving structure of three groups of subjects. All the group-level driving structures consisted of group-level driving nodes, group-level driving edges, the group-level controlled nodes. (**a**) group-level driving structure of NC; (**b**) group-level driving structure of MCI; (**c**) group-level driving structure of AD.

Group-level driving structures differed among the NC, MCI, and AD groups (Figure 3 and Table 2). In NC, driving nodes were mainly distributed in the DMN, VAN, and LIM. In MCI, driving nodes were mainly located in the DMN, SMN, and VAN.; compared with NC, additional SMN driving nodes included PreCG.L, TTG.L, and PoCG.R. In AD, driving nodes were distributed across the DMN, SMN, VAN, and FPN. Notably, cMFG.R in the FPN was identified as a driving node in AD but not in NC or MCI.

Across the three groups, driving nodes were consistently observed in regions belonging to the DMN, suggesting that this network may be commonly involved in the control organization of directed brain effective networks. However, the spatial distribution of driving nodes differed among groups, indicating disease-related redistribution of driving structures from NC to MCI and AD.

### 3.2. Altered Controllability at the Global and RSN Levels

**Figure 4:**
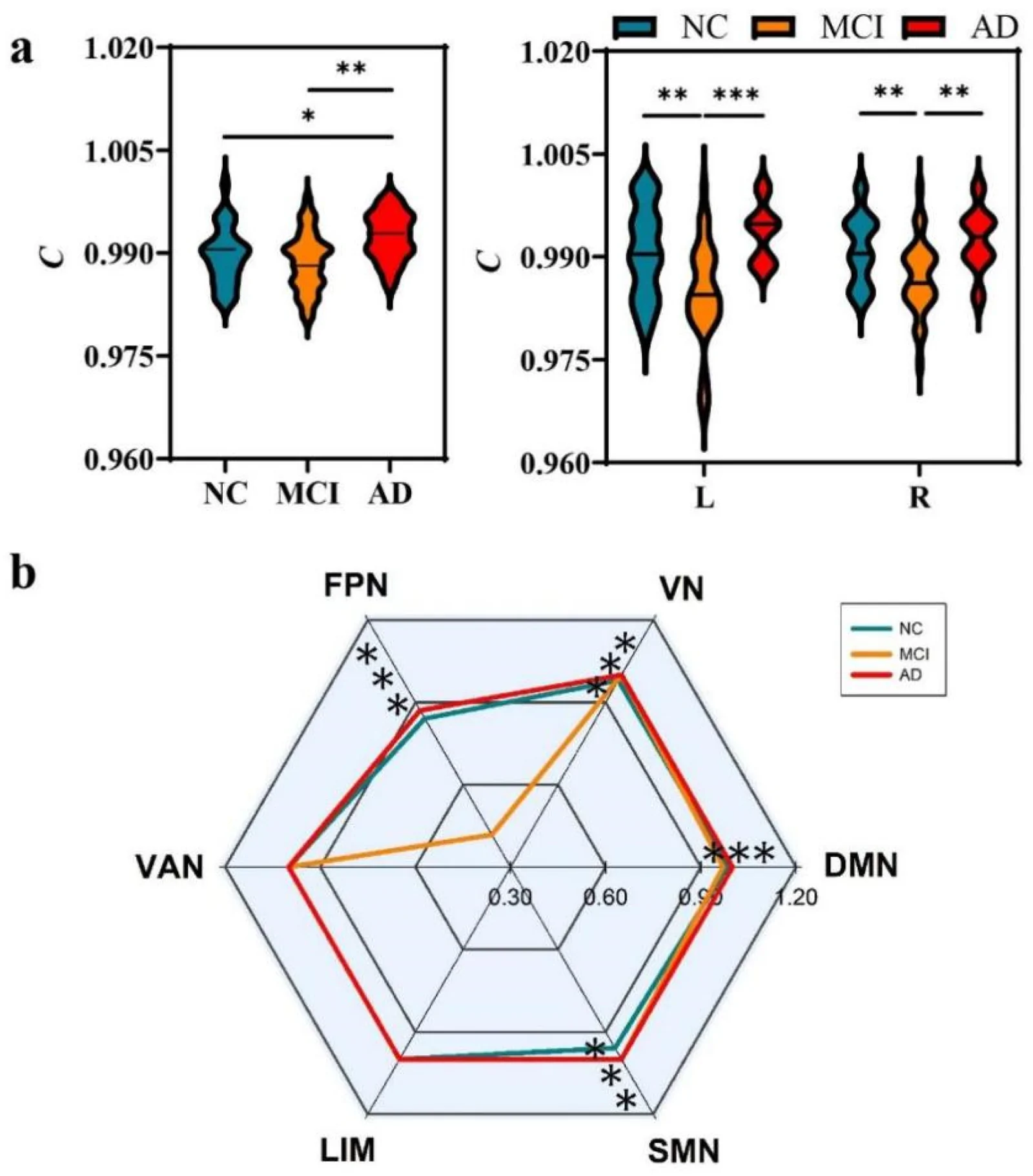
Group differences in controllability index *C* at the global and RSN levels. (**a**) Whole-brain and hemispheric indices. (**b**) Indices for the six RSNs. Asterisks indicate Bonferroni-adjusted pairwise *p* values: * (0.01 < *p* < 0.05), ** (0.001 < *p* < 0.01), and *** (*p* < 0.001). SMN, somatosensory and motor network; DMN, default mode network; VN, visual network; FPN, frontoparietal network; VAN, ventral attention network; LIM, limbic network.

Controllability index was calculated at the whole-brain, hemispheric, and RSN levels. Whole-brain *C* differed among the NC, MCI, and AD groups, decreasing from NC to MCI and increasing from MCI to AD. AD showed a higher whole-brain *C* than NC (*p* < 0.05) and MCI (*p* < 0.01). A similar nonmonotonic pattern was observed in both hemispheres (Figure 4a).

At the RSN level (Figure 4b), the DMN and FPN showed changing patterns similar to the whole-brain *C*. Specifically, *C* in the DMN decreased from NC to MCI and then increased from MCI to AD, with AD showing higher *C* than both NC and MCI. A similar pattern was also observed in the FPN. In contrast, the SMN and VN showed different changing trends, with *C* increasing significantly from NC to MCI or AD. These findings suggest that high-order cognitive networks and sensory-related networks may show different controllability alterations across disease stages.

In addition, the omnibus group effect was significant for whole-brain *C* (F(2, 74) = 6.159, *p* = 0.003, *ηp*^2^ = 0.143). AD showed higher whole-brain *C* than NC (NC–AD mean difference = −0.0036, 95% CI = −0.0065 to −0.0007, Hedges’g = −0.769, Bonferroni-adjusted *p* = 0.044) and MCI (MCI–AD mean difference = −0.0049, 95% CI = −0.0077 to −0.0021, Hedges’g = −1.063, Bonferroni-adjusted *p* = 0.002). Significant omnibus effects were also observed in the DMN and FPN. Complete descriptive and pairwise results are provided in Table S3.

### 3.3. Regional and Hub-Level Controllability Changes

In the exploratory regional omnibus analysis, BH-FDR correction was applied across the 68 cortical-region *p* values. Seventeen regions passed the BH-FDR screening criterion of adjusted *p* < 0.05: cACG.L, ORBinf.L, rMFG.L, SMG.L, FP.L, cACG.R, LOF.R, LING.R, PCL.R, PoCG.R, PCG.R, PreCG.R, PCUN.R, rMFG.R, SFG.R, SPG.R, and STG.R. Complete omnibus results are reported in Table 3. Conditional within-region post hoc comparisons were restricted to these 17 regions and are reported in Table S4. These regional findings are interpreted as exploratory and are not treated as confirmatory biomarkers.

Hub regions were identified from indegree and outdegree characteristics (Table 4). INS.L and INS.R were identified as outdegree hubs across the diagnostic groups, whereas INS.R was identified as a common indegree hub. SFG.L was an outdegree hub in NC, and SFG.R was an outdegree hub in AD.

**Figure 5:**
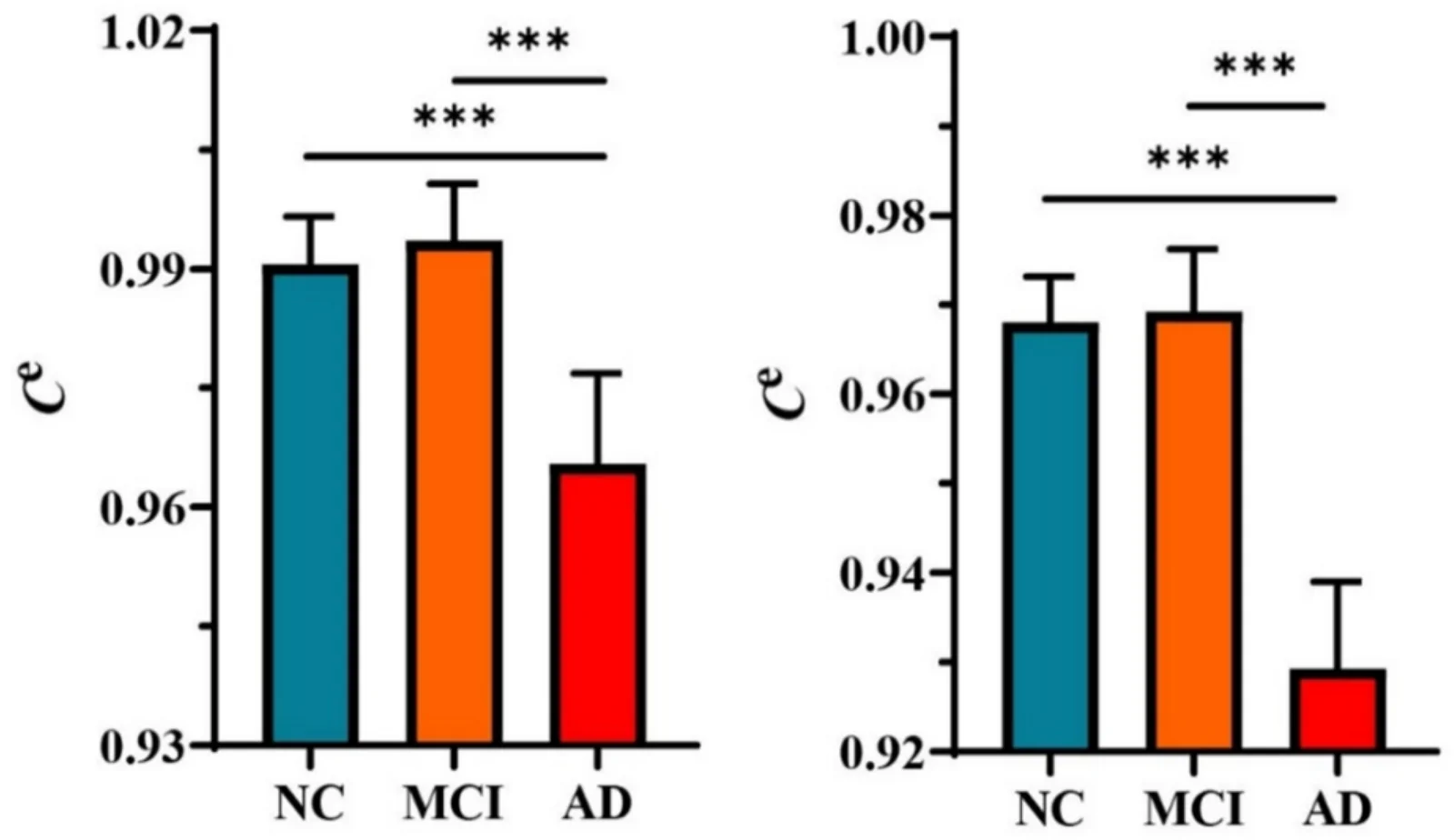
Abnormal changing trend of hubs controllability index *C^e^* of NC, MCI, and AD patients. Abnormal changing trend of controllability index of out-degree hubs (**left**) and in-degree hubs (**right**) of NC, MCI, and AD patients. Asterisks indicate the *p*-values of these differences (Bonferroni correction): *** (*p* < 0.001).

Hub-level *C^e^* showed a group pattern different from that of whole-brain *C* (Figure 5). It increased slightly from NC to MCI and then decreased from MCI to AD. The MCI–AD differences were significant for both outdegree and indegree hubs (*p* < 0.001), as were the NC–AD differences (*p* < 0.001). This dissociation suggests that hub-related control organization may be altered in AD.

### 3.4. Associations Between Controllability, Degree, and Clinical Scores

**Figure 6:**
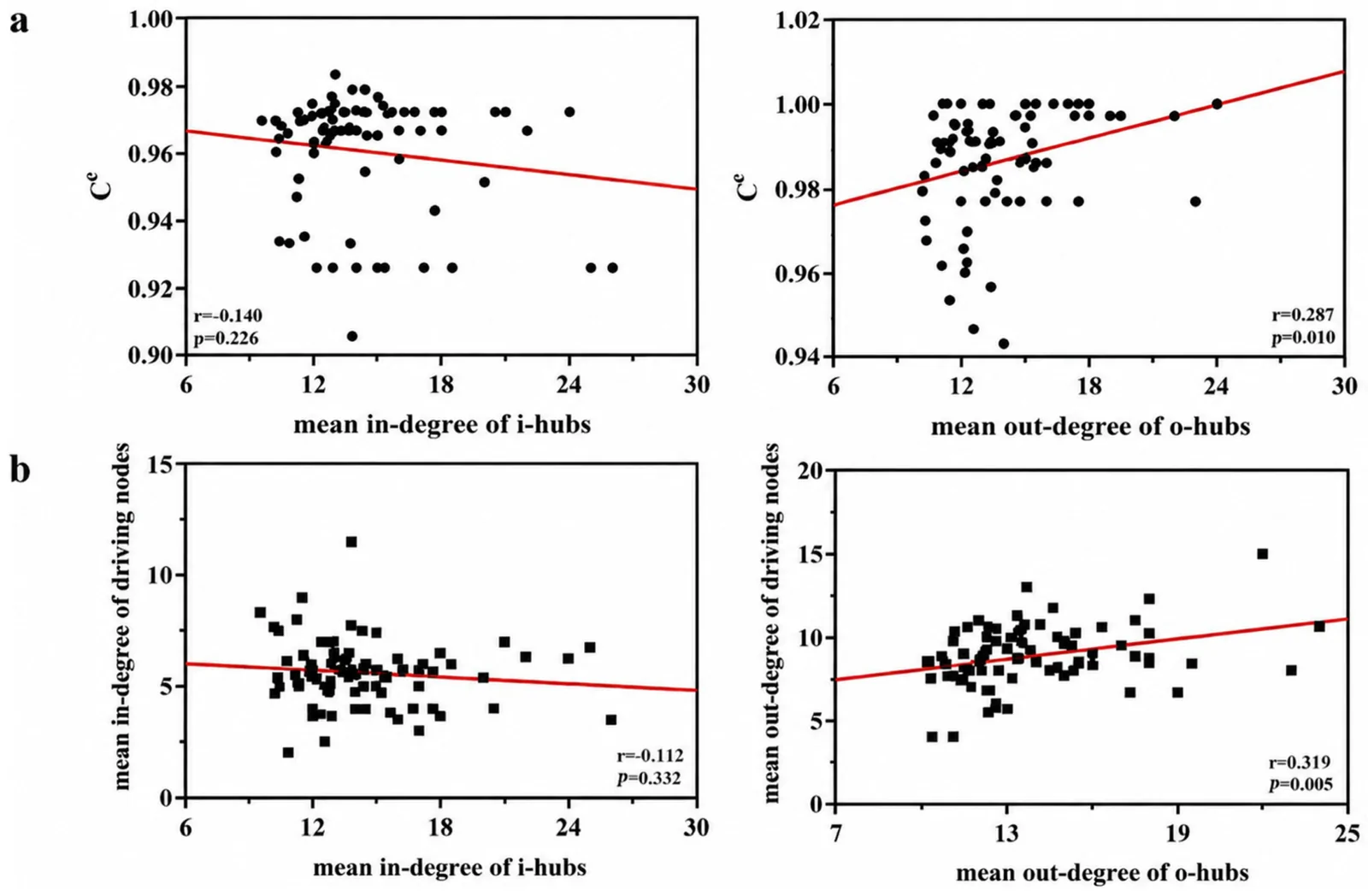
Associations between regional node controllability and node degree. (**a**) Associations of mean hub *C^e^* with the mean indegree and outdegree of the corresponding hubs; each point represents one participant. (**b**) Associations between hub degree and the degree of driving nodes; each point represents one participant. The red lines represent the linear regression lines fitted to the scatter plots, illustrating the overall linear associations between the corresponding variables.

We further examined the relationship between regional node controllability index *C^e^* and network degree (Table S5). *C^e^* showed a weak negative association with indegree, whereas it showed a significant positive association with outdegree. In addition, the outdegree of driving nodes was positively associated with the outdegree of outdegree hubs, suggesting that driving nodes were more likely to correspond to highly outgoing hub regions (Figure 6).

At the whole-brain level, adding the centered linear and squared *C* terms yielded ΔR^2^ values of 0.058, 0.071, and 0.106 for CDR, FAQ, and MMSE, respectively, after adjustment for age and sex. After BH-FDR correction across the three whole-brain models, only the whole-brain–MMSE model met the adjusted criterion. Among the 204 exploratory regional models, three met the regional BH-FDR criterion: SMG.L–FAQ, cACG.R–FAQ, and rMFG.L–MMSE. No regional CDR model survived correction. Because participants were pooled across diagnoses, these associations may reflect diagnostic-group separation and are not interpreted as continuous within-group or diagnosis-independent relationships. A summary is provided in Table 5, and complete statistics are reported in Table S6.

### 3.5. Results: Sensitivity, Reproducibility, and Uncertainty Analyses

Across the six-setting local parameter window, edge-level agreement was low (HCP mean ICC, 0.080–0.185; ADNI within-scan split-half Spearman *ρ*, 0.090–0.170), and the adopted *α* = 0.8, *β* = 0.1 setting did not yield the highest agreement (Table S2). Higher-*β* settings generally showed higher agreement together with greater SC alignment and lower EC rank retention. The numerical whole-brain group ordering, with AD showing the highest and MCI the lowest mean *C*, was observed across all six settings, and the omnibus group effect was significant at each setting (F = 3.90–6.26, *p* = 0.003–0.025, partial *ηp*^2^ = 0.095–0.145) (Table S7A). However, adjusted pairwise evidence was not uniform: the MCI–AD contrast remained significant across all six settings, the NC–MCI contrast did not, and the NC–AD contrast was nonsignificant under several parameter settings.

Across the five exact AD threshold anchors, retained edge counts decreased from 34 to 11 as the occurrence threshold increased from 10% to 35%. Similarity coefficients relative to the 22% structure were descriptive because the thresholded edge sets were nested and the 22% values equaled 1 by definition. Bootstrap Jaccard estimates were 0.58 [0.41, 0.69], 0.67 [0.53, 0.77], 0.72 [0.60, 0.81], 0.75 [0.62, 0.84], and 0.61 [0.44, 0.72]. The intervals at 22% and 28% overlapped, and no threshold was interpreted as uniquely stable or optimal. The numerical whole-brain ordering was again observed across the tested thresholds, and the omnibus group effect was significant at each anchor (F = 5.13–6.16, *p* = 0.003–0.008). However, the NC–MCI comparison was nonsignificant throughout, and the NC–AD comparison did not meet the adjusted criterion at the 35% anchor. These analyses indicate persistence of the numerical whole-brain ordering but not uniform robustness of all pairwise contrasts. Detailed results are presented in Supplementary Figure S3, Tables S7B and S8.

Of the 22 reported nodes, 11 were present at three of the five exact threshold anchors, eight at four anchors, and three at all five anchors. These findings indicate moderate rather than complete cross-threshold stability (Table S9).

The pooled association between *C* and driver-node fraction was negative (*ρ* = −0.579, *p* < 0.001) and remained after adjustment for diagnosis and network density (partial *r* = −0.561, *p* < 0.001). The pooled association with maximum-matching ratio was weak (*ρ* = 0.209, *p* = 0.068) and was absent after adjustment (partial *r* = 0.031, *p* = 0.786). Because *C* and these matching-derived measures originate from the same maximum-matching framework, the analyses are interpreted as descriptive internal consistency checks rather than external validation of *C.* The corresponding unadjusted plots and complete adjusted statistics are presented in Supplementary Figure S4 and Table S10, respectively.

## 4. Discussion

In this study, we constructed structurally constrained brain effective networks and applied structural controllability analysis to examine differences among NC, MCI, and AD groups. By combining anatomical constraints with directed effective connectivity, the framework extends controllability analyses based solely on structural or functional connectivity. The principal findings were as follows: (1) group-level driving structures differed among diagnostic groups; (2) the whole-brain index decreased from NC to MCI and increased from MCI to AD; (3) the DMN and FPN showed patterns similar to the whole brain, whereas the SMN and VN showed different patterns; (4) driving nodes were more closely related to outdegree hubs, while hub-level controllability showed an opposite group pattern; and (5) selected indices were associated with cognitive scores in pooled exploratory analyses.

### 4.1. Disease-Related Redistribution of Driving Structures

The Group-level driving structures differed among the NC, MCI, and AD groups. Within the structural controllability framework, driving nodes represent network components required for controlling the mathematical dynamics of the constructed effective connectivity network. Therefore, these nodes should be interpreted as topologically influential regions rather than experimentally validated causal controllers of brain activity.

Across the three groups, driving nodes were mainly observed in the DMN, suggesting that this network may play an important role in the control organization of directed brain effective networks. This finding is consistent with previous studies showing that the DMN is particularly vulnerable in AD due to its high metabolic demand, hub characteristics, and susceptibility to amyloid-β deposition and tau-related neurodegeneration [36]. The presence of DMN-related driving structures suggests that alterations in this network may influence the organization of directed information interactions.

Compared with NC, MCI showed additional driving nodes in the SMN. Although the SMN is often regarded as a lower-order sensory-motor network, increasing evidence suggests that sensory and motor network alterations may occur in MCI and AD [37]. The presence of SMN driving nodes in MCI may therefore reflect early reorganization of network control architecture during cognitive decline. Rather than indicating that the SMN should be directly targeted for intervention, this result suggests that the SMN may be a candidate network for future studies examining compensatory or disease-related changes in MCI.

In AD, driving nodes were distributed across the DMN, SMN, VAN, and FPN. The involvement of the FPN in AD is particularly relevant because the FPN is closely associated with cognitive control and flexible regulation of brain activity [38]. The appearance of cMFG.R as a driving node in AD may reflect altered control organization in frontal executive systems. However, this interpretation should be considered hypothesis-generating.

Overall, the redistribution of driving nodes from NC to MCI and AD suggests that disease progression is associated with changes in the control architecture of directed brain effective networks. These changes may reflect altered information propagation patterns.

### 4.2. Altered Controllability Patterns Across Global and Network Levels

Structural controllability is dependent on network topology, control objectives, and mathematical assumptions. Therefore, the present controllability index represents one specific characterization of network control properties rather than a comprehensive measure of brain controllability.

At the global level, the whole-brain controllability index decreased from NC to MCI and then increased from MCI to AD. The lower controllability index in MCI may reflect early disruption of directed network organization during cognitive decline [39,40]. In contrast, the increased controllability index in AD should not be interpreted as indicating that the AD brain is easier to modulate clinically. Rather, within the present structural controllability framework, this increase may reflect disease-related reorganization or simplification of directed network topology.

This nonlinear pattern is consistent with the idea that MCI and AD may represent different stages of network reorganization. During MCI, the brain may retain a relatively complex network structure but show reduced efficiency in control organization. During AD, more advanced structural and functional damage may alter the topology of the brain effective network, leading to a different controllability pattern. In addition, compensatory mechanisms and methodological factors related to network construction and controllability modeling may also influence these findings [41]. However, this explanation remains speculative and requires further validation using direct measures of brain atrophy, network density, and longitudinal disease progression.

Several mechanisms may contribute to this nonlinear pattern. First, neurodegenerative processes may selectively affect network connections and reshape the topology underlying controllability estimation [42]. Second, surviving networks may undergo compensatory reorganization to maintain information transmission despite pathological burden [43]. Third, the heterogeneity of MCI and AD, including differences in progression rate and pathological subtype, may contribute to variability in controllability patterns [44]. Therefore, the observed increase from MCI to AD should be interpreted as altered network control organization rather than evidence of enhanced brain function.

At the RSN level, the DMN and FPN showed changing patterns similar to the whole brain, whereas the SMN and VN showed different trends. The DMN and FPN are high-order networks closely related to memory, internal cognition, and executive control. Their similarity to the whole-brain pattern suggests that these networks may contribute strongly to global controllability alterations in MCI and AD. In contrast, the SMN and VN showed different trends, which may indicate that sensory-related networks are involved in disease progression through different control mechanisms. This observation is consistent with network-based models of AD, suggesting that highly connected association networks may be particularly vulnerable to pathological accumulation and disconnection [45].

### 4.3. Hub-Related Controllability and Clinical Associations

Hub regions showed controllability changes that differed from the whole-brain pattern. Specifically, hub-level controllability increased from NC to MCI and then decreased from MCI to AD. This opposite pattern may reflect abnormal reorganization of hub-related control architecture during disease progression. Because hub regions support broad communication across brain networks, changes in their controllability may have important implications for information integration and cognitive function [35].

The insula was identified as a common hub region across groups, and the superior frontal gyrus also appeared as a hub region in specific groups [46,47]. Both regions have been implicated in cognitive control, salience processing, and AD-related network alterations. The reduced hub-level controllability in AD may reflect disruption of these highly connected regions. However, the present findings do not establish whether hub abnormalities are a cause or consequence of disease-related progression.

The association analyses showed that whole-brain and regional node controllability index was related to clinical cognitive scores, including MMSE, CDR, and FAQ. These findings suggest that controllability alterations may be behaviorally relevant [18]. Nevertheless, these associations should be interpreted cautiously because they may partially reflect differences between diagnostic groups rather than continuous individual-level relationships. Therefore, controllability measures should be considered complementary network-level descriptors rather than standalone clinical biomarkers.

Although controllability index represents mathematical properties of network organization, its associations with cognitive measures suggest that it may capture biologically meaningful alterations related to cognitive decline [13]. Unlike traditional clinical assessments that primarily reflect behavioral manifestations, controllability measures provide information regarding alterations in the regulatory organization of brain networks. Therefore, these indices may serve as complementary imaging markers for characterizing disease severity [48] and identifying individuals with abnormal network regulation. Future longitudinal studies are needed to determine whether specific controllability patterns can predict rapid cognitive decline in MCI, monitor disease progression, or evaluate responses to therapeutic interventions. Moreover, altered driving structures identified in this framework may provide potential candidates for neuromodulation studies, although such applications require further experimental validation [49,50].

These findings complement the extensive literature on network disruption in AD, where graph-theoretical studies have consistently reported reduced global efficiency and small-worldness, particularly in the default mode network [51,52]. Unlike static topology measures, controllability theory addresses the brain’s dynamic capacity for state transitions. Our results reveal that this capacity changes nonlinearly across disease stages—global controllability decreased in MCI but increased in AD, whereas hub-level controllability showed the opposite pattern—and that driving nodes redistribute to sensory-motor and frontoparietal networks. These dissociations extend prior connectivity findings [12,18] and demonstrate that controllability analysis provides a unique, dynamics-oriented perspective on network reorganization in AD.

### 4.4. Limitations

Several limitations should be noted. First, the modest and imbalanced sample size is an important limitation. The analyzed ADNI subset comprised 77 participants, including 28 NC, 33 MCI, and 16 AD participants. The small AD group limits precision and increases sensitivity to individual observations, particularly for regional, hub-level, pooled clinical-association, bootstrap, and threshold analyses. Adjustment for age and sex does not overcome this limitation. Moreover, MCI and AD represent clinically and biologically heterogeneous conditions with variable progression trajectories, and the present cross-sectional design cannot determine whether the observed controllability alterations reflect specific disease subtypes, different progression patterns, or general stage-related changes. These analyses are therefore interpreted as exploratory. The whole-brain result is presented as a primary cross-sectional observation that requires replication in larger, balanced, independent, and preferably longitudinal cohorts.

Second, the structural network was limited to 68 cortical ROIs and excluded subcortical structures such as the hippocampus and amygdala. In addition, the single-shell diffusion acquisition and deterministic FACT tractography may be affected by crossing fibers, partial-volume effects, white-matter pathology, and false-positive or false-negative streamline reconstruction. Streamline counts are therefore an indirect and method-dependent proxy for structural connectivity. Future studies should assess whether the findings generalize to cortical–subcortical parcellations and to probabilistic or multi-compartment diffusion models when suitable data are available.

Third, the identified structures and indices depend on methodological choices. Fusion agreement was low in the tested window, and the within-scan ADNI split-half analysis is not a substitute for independent elderly test–retest data. Threshold results are sensitive to nominal anchors and sample size, and similarity coefficients between nested threshold sets are descriptive. Because template construction and normalization were performed within diagnostic groups, direct cross-group comparability may be affected, particularly for regional and hub-level measures. Accordingly, these comparisons are interpreted as exploratory within the present analytical framework. Future confirmation should evaluate whether the findings persist using a common or cross-validated template and common scaling across groups. Maximum matching may be nonunique, and this source of uncertainty should be quantified. *C* is a driver-edge-based descriptive index within this framework rather than an independently validated energy-based controllability measure.

Fourth, hub identification depends on the selected centrality measure and thresholding strategy. Although the percentile-based approach used here provides a reproducible definition of relatively high-centrality regions, alternative thresholds or classification strategies may yield different hub assignments. Future studies using multiple hub identification approaches will help validate the robustness of hub-related findings.

Fifth, MVAR model order may influence effective connectivity estimation. Although *p* = 1 was selected using information criteria, the HCP and ADNI acquisitions differed substantially in temporal resolution, and alternative orders may produce different directed networks. The present results should therefore be interpreted within the selected model specification, and future analyses should evaluate the robustness of the principal findings at additional plausible model orders.

Finally, the structural controllability framework used here represents one mathematical perspective on network regulation. Although driver nodes, driver edges, and controllability index are standard concepts in network control theory, they describe topological properties rather than experimentally demonstrated causal mechanisms. Granger causality captures statistical directional dependencies rather than biological causation, and identified driving structures cannot be directly interpreted as regions whose stimulation would necessarily produce predicted changes in brain function.

## 5. Conclusions

In this study, we constructed structurally constrained brain effective networks and applied a structural controllability framework to investigate abnormalities in driving structures and controllability patterns in MCI and AD. The results showed that the organization of driving structures differed across NC, MCI, and AD groups. Specifically, driving nodes were mainly distributed within the DMN across groups, while MCI showed additional involvement of the SMN and AD showed further involvement of the FPN, suggesting a redistribution of network control architecture during disease progression.

At the global level, the whole-brain controllability index decreased from NC to MCI and then increased from MCI to AD, indicating that the control organization of brain effective networks changes nonlinearly across disease stages. At the modular level, the DMN and FPN showed changing patterns similar to the whole brain, whereas the SMN and VN showed different trends, suggesting that high-order cognitive networks and lower-order sensory-motor networks may be differently involved in disease-related network control alterations.

At the regional level, node controllability was negatively associated with indegree and positively associated with outdegree, and driving nodes tended to correspond to outdegree hubs. These findings suggest that brain regions with stronger outgoing connections may play important roles in controlling directed brain effective networks. In contrast to the whole-brain pattern, the controllability index of hub regions increased from NC to MCI and decreased from MCI to AD, which may reflect abnormal changes in hub-related control organization during cognitive decline.

Overall, this study suggests that MCI and AD may be associated with redistribution of driving structures and changes in a driver-edge-based index derived from directed brain effective networks. Across the tested parameter and threshold settings, the numerical whole-brain group ordering was repeatedly observed, whereas edge-level agreement was low and the exact regional node set varied. The findings therefore provide preliminary network-level observations rather than validation of a specific parameter setting, threshold, regional node set, or established controllability construct. The fusion procedure, occurrence threshold, template construction, normalization procedure, and index *C* require further evaluation using independent test–retest data, alternative template-construction and normalization strategies and larger independent cohorts.

## Figures and Tables

**Table 1:** Demographic and clinical characteristics of the ADNI participants.

Variable	NC (n = 28)	MCI (n = 33)	AD (n = 16)	Test Statistic	*p*-Value	Effect Size
Sex (M/F)	13/15	17/16	8/8	χ^2^(2) = 0.160	0.923 ^a^	Cramér’s V = 0.046
Age (years)	73.18 ± 7.96	76.53 ± 9.48	74.55 ± 9.15	F(2, 74) = 1.116	0.333 ^b^	*η*^2^ = 0.029
CDR	0.00 ± 0.00	0.50 ± 0.18	0.81 ± 0.40	F(2, 74) = 80.488	<0.001 ^b^	*η*^2^ = 0.685
FAQ	0.03 ± 0.19	4.36 ± 4.72	15.50 ± 7.48	F(2, 74) = 58.803	<0.001 ^b^	*η*^2^ = 0.614
MMSE	28.79 ± 1.72	26.79 ± 3.12	22.63 ± 2.36	F(2, 74) = 30.185	<0.001 ^b^	*η*^2^ = 0.449

Data are presented as mean ± standard deviation (SD). ^a^ Pearson chi-square test. ^b^ One-way analysis of variance (ANOVA). Bonferroni-corrected post hoc comparisons were used for clinical scores.

**Table 2:** Group-level driving regions by diagnostic group.

RSN	NC	MCI	AD
DMN	BSTS.L	IPL.R	IPL.R
	SFG.L	MTG.R	MTG.R
	MTG.R	PCUN.R	
SMN		PreCG.L	STG.L
		TTG.L	
		PoCG.R	
VN	LING.R		
VAN	cACG.R	INS.L	SMG.R
	INS.R	cACG.R	
		SMG.L	
FPN			cMFG.R
LIM	TP.L		
	LOF.L		

**Table 3:** Exploratory omnibus analysis of regional node-index differences across diagnostic groups.

ROI	NC (n = 28) Mean ± SD	MCI (n = 33) Mean ± SD	AD (n = 16) Mean ± SD	F(2, 74)	Omnibus *p*-Value	BH-Adjusted *p*-Value	Partial *ηp*^2^
BSTS.L	0.69801 ± 0.04954	0.70502 ± 0.04291	0.68222 ± 0.04864	1.290	0.281	0.479	0.034
cACG.L	0.81679 ± 0.04431	0.81469 ± 0.04674	0.85878 ± 0.05712	5.091	0.008	0.038	0.121
cMFG.L	0.64401 ± 0.05087	0.65153 ± 0.05770	0.64940 ± 0.04404	0.158	0.854	0.907	0.004
CUN.L	0.72062 ± 0.04790	0.70808 ± 0.06028	0.71201 ± 0.05148	0.411	0.664	0.754	0.011
OLF.L	0.63261 ± 0.05856	0.62566 ± 0.05722	0.63157 ± 0.07294	0.110	0.896	0.923	0.003
FFG.L	0.66674 ± 0.05537	0.68480 ± 0.06166	0.67677 ± 0.05568	0.728	0.486	0.615	0.019
IPL.L	0.60670 ± 0.05544	0.62959 ± 0.05552	0.62589 ± 0.05252	1.414	0.250	0.479	0.037
ITG.L	0.71156 ± 0.07154	0.72816 ± 0.06106	0.74923 ± 0.06410	1.694	0.191	0.450	0.044
ICG.L	0.78637 ± 0.03793	0.77039 ± 0.03652	0.78854 ± 0.03657	1.949	0.150	0.418	0.050
LOG.L	0.69329 ± 0.04497	0.68776 ± 0.04344	0.69074 ± 0.04560	0.118	0.889	0.923	0.003
LOF.L	0.73664 ± 0.05706	0.74242 ± 0.06065	0.75416 ± 0.05835	0.451	0.639	0.754	0.012
LING.L	0.77982 ± 0.05906	0.78657 ± 0.06354	0.76196 ± 0.06717	0.834	0.438	0.588	0.022
MOF.L	0.79895 ± 0.04831	0.81337 ± 0.04459	0.78904 ± 0.03790	1.780	0.176	0.443	0.046
MTG.L	0.63197 ± 0.05639	0.63368 ± 0.05731	0.65681 ± 0.06728	1.043	0.358	0.529	0.027
PHG.L	0.79177 ± 0.06332	0.78364 ± 0.05380	0.80691 ± 0.06328	0.828	0.441	0.588	0.022
PCL.L	0.70776 ± 0.05391	0.72047 ± 0.07255	0.67933 ± 0.07296	2.067	0.134	0.418	0.053
pOPER.L	0.63911 ± 0.03274	0.63554 ± 0.04421	0.64710 ± 0.03381	0.490	0.615	0.746	0.013
ORBinf.L	0.75066 ± 0.05487	0.74796 ± 0.05282	0.80459 ± 0.07036	5.893	0.004	0.020	0.137
IFGtriang.L	0.75783 ± 0.03855	0.74938 ± 0.03363	0.75136 ± 0.03660	0.433	0.650	0.754	0.012
periCAG.L	0.72535 ± 0.05960	0.74676 ± 0.05246	0.74271 ± 0.05122	1.220	0.301	0.489	0.032
PoCG.L	0.78400 ± 0.04105	0.79776 ± 0.04680	0.80701 ± 0.04202	1.547	0.220	0.479	0.040
PCG.L	0.64393 ± 0.04858	0.63972 ± 0.06961	0.63239 ± 0.06283	0.180	0.835	0.902	0.005
PreCG.L	0.66163 ± 0.06905	0.68424 ± 0.05486	0.68220 ± 0.06091	1.137	0.326	0.496	0.030
PCUN.L	0.73946 ± 0.06220	0.74610 ± 0.05740	0.74278 ± 0.06338	0.091	0.913	0.925	0.002
rACG.L	0.69078 ± 0.04485	0.69126 ± 0.05113	0.71434 ± 0.05309	1.413	0.250	0.479	0.037
rMFG.L	0.76825 ± 0.05358	0.76455 ± 0.04910	0.84220 ± 0.05139	13.966	<0.001	<0.001	0.274
SFG.L	0.65843 ± 0.04650	0.65443 ± 0.04439	0.65393 ± 0.04110	0.078	0.925	0.925	0.002
SPG.L	0.77924 ± 0.04009	0.76593 ± 0.04061	0.78270 ± 0.03659	1.312	0.276	0.479	0.034
STG.L	0.61684 ± 0.04518	0.63494 ± 0.05020	0.62986 ± 0.04500	1.135	0.327	0.496	0.030
SMG.L	0.71843 ± 0.05270	0.66654 ± 0.04339	0.77338 ± 0.03976	29.831	<0.001	<0.001	0.446
FP.L	0.80418 ± 0.04833	0.80132 ± 0.04360	0.86151 ± 0.04615	10.459	<0.001	<0.001	0.220
TP.L	0.64341 ± 0.04918	0.65810 ± 0.05229	0.65646 ± 0.04280	0.739	0.481	0.615	0.020
TTG.L	0.70269 ± 0.04380	0.72148 ± 0.04759	0.72842 ± 0.04502	2.013	0.141	0.418	0.052
INS.L	0.66612 ± 0.04420	0.64948 ± 0.03639	0.65858 ± 0.03211	1.413	0.250	0.479	0.037
BSTS.R	0.80178 ± 0.05440	0.82376 ± 0.05320	0.81158 ± 0.04166	1.388	0.256	0.479	0.036
cACG.R	0.61170 ± 0.06740	0.60589 ± 0.05594	0.72794 ± 0.06935	22.724	<0.001	<0.001	0.380
cMFG.R	0.78539 ± 0.05402	0.81047 ± 0.05497	0.81011 ± 0.05005	1.929	0.152	0.418	0.050
CUN.R	0.69563 ± 0.06456	0.71063 ± 0.05415	0.72601 ± 0.06034	1.374	0.259	0.479	0.036
OLF.R	0.63267 ± 0.06533	0.61151 ± 0.06462	0.60491 ± 0.06334	1.216	0.302	0.489	0.032
FFG.R	0.64780 ± 0.04941	0.65553 ± 0.04783	0.66528 ± 0.03818	0.723	0.489	0.615	0.019
IPL.R	0.79441 ± 0.05607	0.77442 ± 0.04746	0.78855 ± 0.04137	1.289	0.282	0.479	0.034
ITG.R	0.64173 ± 0.04416	0.65189 ± 0.05122	0.66358 ± 0.04129	1.131	0.328	0.496	0.030
ICG.R	0.70341 ± 0.06791	0.69483 ± 0.05307	0.69018 ± 0.05105	0.299	0.742	0.814	0.008
LOG.R	0.78782 ± 0.03435	0.79960 ± 0.03665	0.78905 ± 0.04085	0.903	0.410	0.588	0.024
LOF.R	0.67162 ± 0.04387	0.69111 ± 0.03867	0.73010 ± 0.03390	11.025	<0.001	<0.001	0.230
LING.R	0.70791 ± 0.04153	0.70587 ± 0.05304	0.74864 ± 0.05008	4.725	0.012	0.047	0.113
MOF.R	0.81529 ± 0.03846	0.81045 ± 0.04536	0.83643 ± 0.05187	1.900	0.157	0.418	0.049
MTG.R	0.81356 ± 0.04409	0.80324 ± 0.05293	0.79720 ± 0.03933	0.691	0.504	0.624	0.018
PHG.R	0.64122 ± 0.05464	0.61611 ± 0.05361	0.61860 ± 0.06292	1.688	0.192	0.450	0.044
PCL.R	0.63332 ± 0.05479	0.64996 ± 0.04723	0.68324 ± 0.05297	4.833	0.011	0.045	0.116
pOPER.R	0.78576 ± 0.03641	0.76566 ± 0.04740	0.77538 ± 0.04550	1.633	0.202	0.459	0.042
ORBinf.R	0.64010 ± 0.04541	0.62574 ± 0.04155	0.61860 ± 0.05112	1.360	0.263	0.479	0.035
IFGtriang.R	0.65300 ± 0.05273	0.66586 ± 0.04693	0.67277 ± 0.05642	0.878	0.420	0.588	0.023
periCAG.R	0.67968 ± 0.04497	0.68510 ± 0.03821	0.68982 ± 0.04424	0.312	0.733	0.814	0.008
PoCG.R	0.68049 ± 0.07273	0.63077 ± 0.07135	0.71492 ± 0.05578	8.937	<0.001	0.002	0.195
PCG.R	0.73480 ± 0.07094	0.73023 ± 0.06058	0.82608 ± 0.07358	12.359	<0.001	<0.001	0.250
PreCG.R	0.65150 ± 0.05124	0.60485 ± 0.05006	0.70089 ± 0.05173	20.036	<0.001	<0.001	0.351
PCUN.R	0.71991 ± 0.06352	0.66540 ± 0.04878	0.71704 ± 0.05368	8.749	<0.001	0.002	0.191
rACG.R	0.71883 ± 0.06898	0.74293 ± 0.05386	0.75606 ± 0.06674	2.083	0.132	0.418	0.053
rMFG.R	0.81791 ± 0.03955	0.81565 ± 0.03919	0.86304 ± 0.03462	9.241	<0.001	0.002	0.200
SFG.R	0.82008 ± 0.03300	0.79449 ± 0.03478	0.84717 ± 0.04389	11.887	<0.001	<0.001	0.243
SPG.R	0.78005 ± 0.04926	0.77655 ± 0.04165	0.85011 ± 0.04269	16.413	<0.001	<0.001	0.307
STG.R	0.69525 ± 0.04405	0.69157 ± 0.05974	0.76880 ± 0.04865	13.279	<0.001	<0.001	0.264
SMG.R	0.66789 ± 0.04479	0.67613 ± 0.03647	0.66770 ± 0.03713	0.410	0.665	0.754	0.011
FP.R	0.80495 ± 0.04530	0.81900 ± 0.04667	0.83342 ± 0.04344	2.052	0.136	0.418	0.053
TP.R	0.63209 ± 0.03994	0.64880 ± 0.04622	0.62618 ± 0.04295	1.879	0.160	0.418	0.048
TTG.R	0.63403 ± 0.04240	0.62769 ± 0.04956	0.61546 ± 0.04324	0.839	0.436	0.588	0.022
INS.R	0.74461 ± 0.05866	0.77251 ± 0.05617	0.75061 ± 0.04979	2.055	0.135	0.418	0.053

The BH procedure was applied across 68 omnibus tests. Statistical significance was defined by BH-adjusted *p* < 0.05. *ηp*^2^ denotes partial eta-squared.

**Table 4:** Hub regions identified in each diagnostic group.

NC		MCI		AD	
outdegree HUB	indegree HUB	outdegree HUB	indegree HUB	outdegree HUB	indegree HUB
SFG.L *	INS.L	INS.L *	INS.L *	INS	INS.R
INS.L	INS.R *	INS.R	INS.R	SFG.R	
INS.R *	SFG.R		SFG.R		

* means this node is a driving node.

**Table 5:** Incremental age- and sex-adjusted associations between controllability and clinical scores from hierarchical second-order polynomial models.

Outcome	Clinical Score	ΔR^2^	F-Change (2,72)	*p*	BH-Adjusted *p*
Whole brain	CDR	0.058	2.354	0.1023	0.1023
Whole brain	FAQ	0.071	2.976	0.0573	0.0860
Whole brain	MMSE	0.106	4.717	0.0119	0.0357 *
SMG.L	FAQ	0.198	9.738	0.0002	0.0311 *
cACG.R	FAQ	0.180	8.640	0.0004	0.0311 *
rMFG.L	MMSE	0.176	8.574	0.0005	0.0311 *

* BH-adjusted (0.01 < *p* < 0.05).

## Data Availability

All data used in this study were obtained from the Human Connectome Project (HCP) Retest data release (http://www.humanconnectome.org/ (accessed on 29 July 2026)) and the Alzheimer’s Disease Neuroimaging Initiative (ADNI) database (http://adni.loni.usc.edu/ (accessed on 29 July 2026)). The ADNI database is a de-identified research database that is publicly shared with authorized researchers through the LONI Image and Data Archive (IDA). Researchers can request access through the official ADNI website upon registration and agreement to the data use terms.

## Supplementary Materials

The following supporting information can be downloaded at: https://www.mdpi.com/article/10.3390/brainsci16080820/s1, Figure S1: MVAR model error noise based on AIC and BIC calculation; Figure S2: Reproducibility and sensitivity of the SC–EC fusion parameters across the α–β grid; Figure S3: Quantitative sensitivity analysis of the AD driving structure at five exact nominal occurrence-threshold anchors. (a) Retained edge count and the minimum occurrence count implied by each threshold (AD n = 16); (b) Jaccard and Dice similarity to the 22% reference; (c) bootstrap Jaccard estimates with intervals; Figure S4: Descriptive consistency checks between *C* and matching-derived measures; Table S1: Brain regio n information; Table S2: Local sensitivity window for the fusion parameters; Table S3A: Descriptive statistics and omnibus group effects for global, hemispheric, RSN, and hub-level indices; Table S3B: Pairwise group comparisons for global, hemispheric, RSN, and hub-level indices; Table S4: Pairwise effect sizes for regional index differences; Table S5: Correlation between *C^e^* and nodes degree; Table S6: Complete hierarchical second-order polynomial regression results; Table S7A: Whole-brain *C* across the six-setting local sensitivity window; Table S7B: Whole-brain *C* across five exact nominal threshold anchors; Table S8: AD threshold sensitivity at five exact nominal anchors; Table S9: Cross-threshold prevalence of the reported driving nodes; Table S10: Descriptive associations of *C* with matching-derived measures.

## Abbreviations

The following abbreviations are used in this manuscript:

AD	Alzheimer’s Disease
ADNI	Alzheimer’s Disease Neuroimaging Initiative
AIC	Akaike Information Criterion
ANOVA	Analysis of Variance
BIC	Bayesian Information Criterion
BH	Benjamini–Hochberg
CDR	Clinical Dementia Rating
DTI	Diffusion Tensor Imaging
DMN	Default Mode Network
FACT	Fiber Assignment by Continuous Tracking
FAQ	Functional Activities Questionnaire
FDR	False Discovery Rate
fMRI	functional Magnetic Resonance Imaging
GCA	Granger Causality Analysis
HCP	Human Connectome Project
ICC	Intraclass Correlation Coefficient
MCI	Mild Cognitive Impairment
MMSE	Mini-Mental State Examination
MRI	Magnetic Resonance Imaging
MVAR	MultiVariate AutoRegressive
NC	Normal Control
rs-fMRI	Resting-state fMRI
ROI	Region of Interest
RSN	Resting-State Network
