# Supplementary material for: Altered Driving Structures and Controllability Patterns of Brain Effective Networks in Mild Cognitive Impairment and Alzheimer’s Disease
Source: Brain Sci. 2026 Jul 31;16(8):820. doi: 10.3390/brainsci16080820 (PMC13511545; doi:10.3390/brainsci16080820)
Supplement: Supplementary file 1 [file brainsci-16-00820-s001.zip › brainsci-4408683-supplementary.pdf]

Article

Altered Driving Structures and Controllability Patterns of Brain Effective Networks in Mild Cognitive Impairment and Alzheimer's Disease

Jiayue Xue[1,*], Xinyi Yan[1], Jing Wei[1], Min Gao[1], Ke Liang[2]

[1] School of Information, Shanxi University of Finance and Economics, Taiyuan 030006, China.;
[1] School of Information, Shanxi University of Finance and Economics, Taiyuan 030006, China.; 252125500057@stu.sxufe.edu.cn
[1] School of Information, Shanxi University of Finance and Economics, Taiyuan 030006, China.; 20141032@sxufe.edu.cn
[1] School of Information, Shanxi University of Finance and Economics, Taiyuan 030006, China.; 20241047@sxufe.edu.cn
[2] School of Statistics, Shanxi University of Finance and Economics, Taiyuan 030006, China; 20241003@sxufe.edu.cn
* Correspondence: 20241004@sxufe.edu.cn;

Academic Editor: Firstname Lastname

Received: date
Revised: date
Accepted: date
Published: date

Copyright: © 2026 by the authors. Submitted for possible open access publication under the terms and conditions of the Creative Commons Attribution (CC BY) license.

Figure S1. MVAR model error noise based on AIC and BIC calculation.

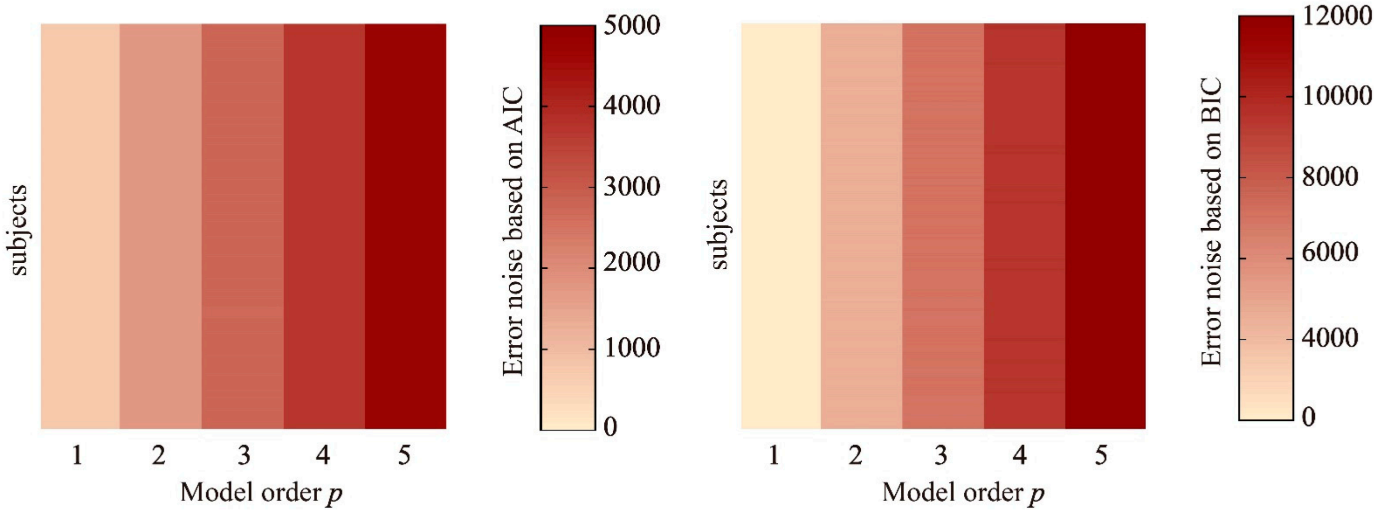

Figure S2. Reproducibility and sensitivity of the SC–EC fusion parameters across the α–β grid.

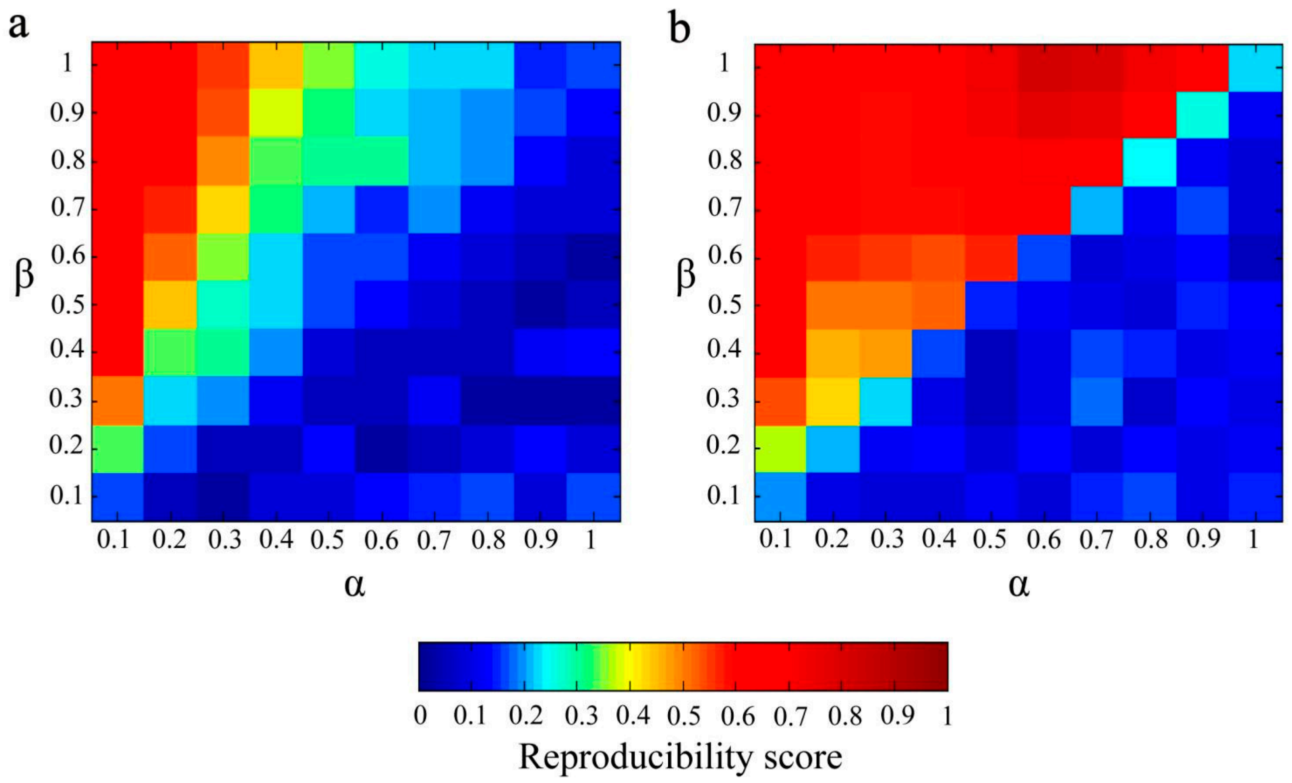

Figure S3. Quantitative sensitivity analysis of the AD driving structure at five exact nominal occurrence-threshold anchors. (a) Retained edge count and the minimum occurrence count implied by each threshold (AD n=16); (b) Jaccard and Dice similarity to the 22% reference; (c) bootstrap Jaccard estimates with intervals.

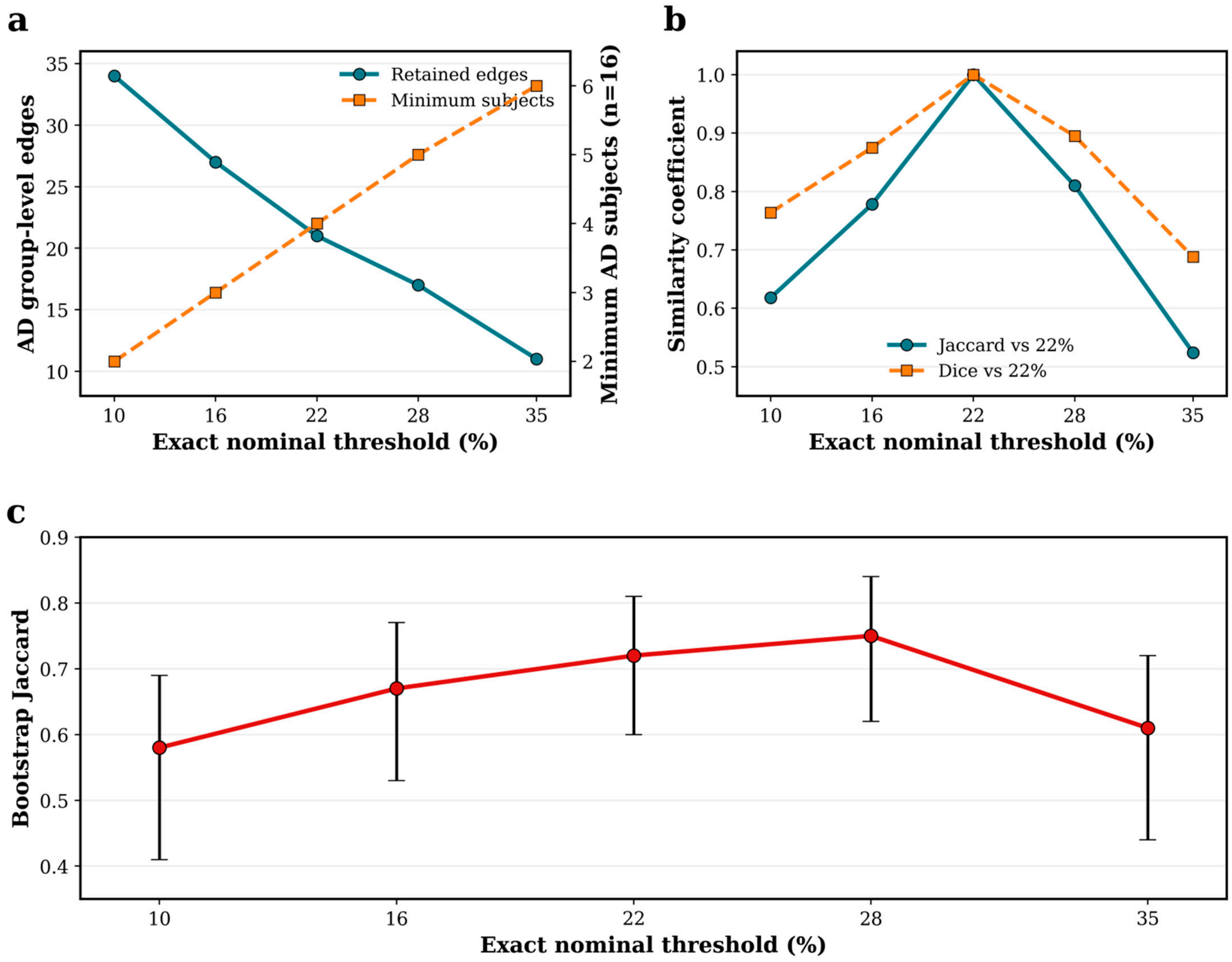

Figure S4. Descriptive consistency checks between C and matching-derived measures.

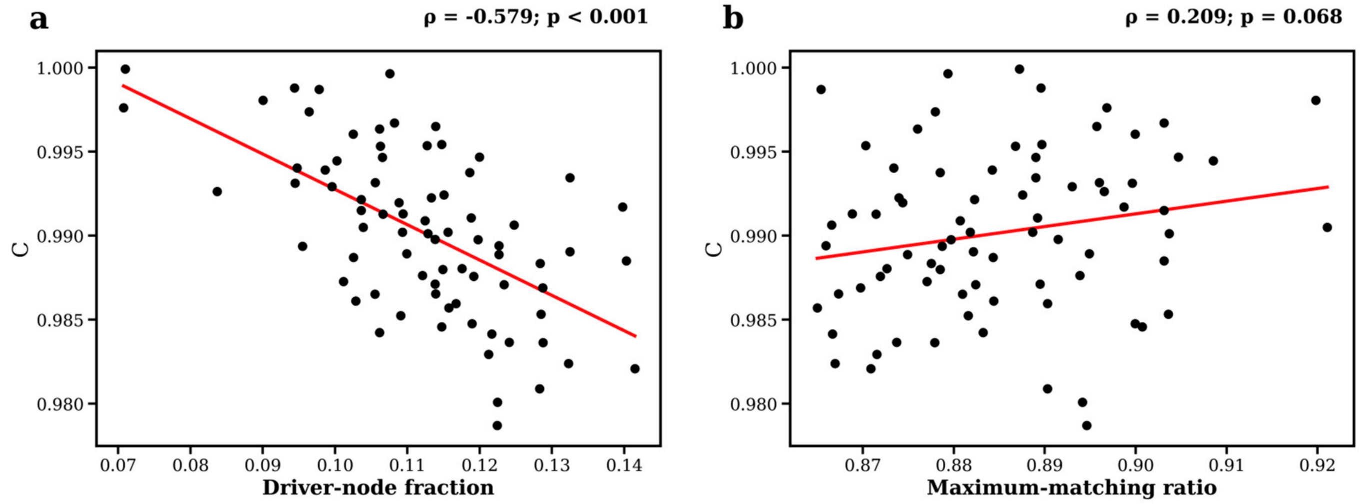

Table S1. Brain regio n information.

RSN	Name	Abbreviation
Somatosensory and Motor Network (SMN)	Paracentral lobule	PCL
	Precentral gyrus	PreCG
	Postcentral gyrus	PoCG
	Superior temporal gyrus	STG
	Transverse temporal gyrus	TTG
Default Mode Network (DMN)	Bankssts	BSTS
	Inferior parietal gyrus	IPL
	Isthmus cingulate gyrus	ICG
	Middle temporal gyrus	MTG
	Parahippocampal gyrus	PHG
	Inferior frontal gyrus orbital part	ORBinf
	Posterior cingulate gyrus	PCG
	Precuneus	PCUN
	Rostral anterior cingulate gyrus	rACG
	Superior frontal gyrus	SFG
Visual Network (VN)	Cuneus	CUN
	Fusiform gyrus	FFG
	Lateral occipital gyrus	LOG
	Lingual gyrus	LING
	Pericalcarine gyrus	periCAG
Fronto-Parietal Network (FPN)	Caudal middle frontal gyrus	cMFG
	Rostral middle frontal gyrus	rMFG
	Superior parietal gyrus	SPG
Ventral Attention Network (VAN)	Caudal anterior cingulate gyrus	cACG
	Parsopercularis	pOPER
	Inferior frontal gyrus, triangular part	IFGtriang
	Supramarginal gyrus	SMG
	Insula	INS
Limbic system (LIM)	Olfactory cortex	OLF
	Inferior temporal gyrus	ITG
	Lateral orbitofrontal	LOF
	Medial orbitofrontal	MOF
	Frontalpole	FP
	Temporalpole	TP

Table S2. Local sensitivity window for the fusion parameters.

α	β	HCP mean ICC	ADNI split-half ϱ	EC retention ϱ	SC alignment ϱ
0.7	0.1	0.130	0.110	0.840	0.190
0.8	0.1	0.110	0.090	0.890	0.160
0.9	0.1	0.080	0.120	0.930	0.140
0.7	0.2	0.185	0.170	0.790	0.300
0.8	0.2	0.170	0.150	0.820	0.270
0.9	0.2	0.120	0.100	0.870	0.230

Table S3A. Descriptive statistics and omnibus group effects for global, hemispheric, RSN, and hub-level indices.

Outcome	NC (n = 28), mean ± SD (95% CI)	MCI (n = 33), mean ± SD (95% CI)	AD (n = 16), mean ± SD (95% CI)	F(2, 74)	*p*-value	Partial ηp^2
Whole brain	0.9900 ± 0.0047 (0.9882–0.9918)	0.9887 ± 0.0046 (0.9871–0.9903)	0.9936 ± 0.0044 (0.9913–0.9959)	6.159	0.003	0.143
Left hemisphere	0.9897 ± 0.0049 (0.9878–0.9916)	0.9883 ± 0.0048 (0.9866–0.9900)	0.9934 ± 0.0045 (0.9910–0.9958)	6.166	0.003	0.143
Right hemisphere	0.9902 ± 0.0048 (0.9883–0.9921)	0.9889 ± 0.0047 (0.9872–0.9906)	0.9939 ± 0.0044 (0.9916–0.9962)	6.200	0.003	0.144
DMN	0.7040 ± 0.0530 (0.6834–0.7246)	0.6820 ± 0.0490 (0.6646–0.6994)	0.7440 ± 0.0460 (0.7195–0.7685)	8.322	<0.001	0.184
FPN	0.7160 ± 0.0450 (0.6986–0.7334)	0.6990 ± 0.0480 (0.6820–0.7160)	0.7520 ± 0.0430 (0.7291–0.7749)	7.173	0.001	0.162
SMN	0.6810 ± 0.0560 (0.6593–0.7027)	0.7160 ± 0.0520 (0.6976–0.7344)	0.7240 ± 0.0500 (0.6974–0.7506)	4.591	0.013	0.110
VN	0.6550 ± 0.0600 (0.6317–0.6783)	0.6910 ± 0.0550 (0.6715–0.7105)	0.7040 ± 0.0520 (0.6763–0.7317)	4.840	0.011	0.116
VAN	0.6920 ± 0.0540 (0.6711–0.7129)	0.6870 ± 0.0560 (0.6671–0.7069)	0.7010 ± 0.0500 (0.6744–0.7276)	0.361	0.698	0.010
LIM	0.6730 ± 0.0580 (0.6505–0.6955)	0.6650 ± 0.0530 (0.6462–0.6838)	0.6810 ± 0.0500 (0.6544–0.7076)	0.491	0.614	0.013
Outdegree hubs	0.9898 ± 0.0067 (0.9872–0.9924)	0.9929 ± 0.0078 (0.9901–0.9957)	0.9648 ± 0.0119 (0.9585–0.9711)	64.203	<0.001	0.634
Indegree hubs	0.9675 ± 0.0057 (0.9653–0.9697)	0.9687 ± 0.0075 (0.9660–0.9714)	0.9287 ± 0.0103 (0.9232–0.9342)	171.171	<0.001	0.822

Table S3B. Pairwise group comparisons for global, hemispheric, RSN, and hub-level indices.

Outcome	Comparison	Mean difference	95% CI	Hedges' g	95% CI for g	Bonferroni-corrected *p*-value
Whole brain	NC–MCI	0.0013	-0.0011 to 0.0037	0.276	-0.230 to 0.782	0.824
Whole brain	NC–AD	-0.0036	-0.0065 to -0.0007	-0.769	-1.405 to -0.133	0.044
Whole brain	MCI–AD	-0.0049	-0.0077 to -0.0021	-1.063	-1.697 to -0.428	0.002
Left hemisphere	NC–MCI	0.0014	-0.0010 to 0.0038	0.285	-0.221 to 0.791	0.773
Left hemisphere	NC–AD	-0.0037	-0.0067 to -0.0007	-0.763	-1.399 to -0.128	0.047
Left hemisphere	MCI–AD	-0.0051	-0.0080 to -0.0022	-1.066	-1.701 to -0.431	0.002
Right hemisphere	NC–MCI	0.0013	-0.0011 to 0.0037	0.270	-0.236 to 0.776	0.849
Right hemisphere	NC–AD	-0.0037	-0.0066 to -0.0008	-0.780	-1.416 to -0.143	0.041
Right hemisphere	MCI–AD	-0.0050	-0.0078 to -0.0022	-1.068	-1.703 to -0.433	0.002
DMN	NC–MCI	0.0220	-0.0036 to	0.427	-0.082 to	0.271

Outcome	Comparison	Mean difference	95% CI	Hedges′ g	95% CI for g	Bonferroni-corrected *p*-value
			0.0476		0.936	
DMN	NC–AD	-0.0400	-0.0712 to -0.0088	-0.776	-1.412 to -0.140	0.038
DMN	MCI–AD	-0.0620	-0.0923 to -0.0317	-1.269	-1.919 to -0.619	<0.001
FPN	NC–MCI	0.0170	-0.0065 to 0.0405	0.360	-0.148 to 0.868	0.462
FPN	NC–AD	-0.0360	-0.0647 to -0.0073	-0.798	-1.436 to -0.161	0.044
FPN	MCI–AD	-0.0530	-0.0809 to -0.0251	-1.122	-1.761 to -0.484	<0.001
SMN	NC–MCI	-0.0350	-0.0622 to -0.0078	-0.641	-1.158 to -0.125	0.037
SMN	NC–AD	-0.0430	-0.0762 to -0.0098	-0.783	-1.420 to -0.146	0.035
SMN	MCI–AD	-0.0080	-0.0402 to 0.0242	-0.153	-0.751 to 0.445	1.000
VN	NC–MCI	-0.0360	-0.0648 to -0.0072	-0.620	-1.136 to -0.104	0.045
VN	NC–AD	-0.0490	-0.0842 to -0.0138	-0.840	-1.480 to -0.200	0.021
VN	MCI–AD	-0.0130	-0.0472 to 0.0212	-0.237	-0.836 to 0.362	1.000
VAN	NC–MCI	0.0050	-0.0227 to 0.0327	0.090	-0.414 to 0.593	1.000
VAN	NC–AD	-0.0090	-0.0428 to 0.0248	-0.168	-0.783 to 0.447	1.000
VAN	MCI–AD	-0.0140	-0.0468 to 0.0188	-0.254	-0.854 to 0.345	1.000
LIM	NC–MCI	0.0080	-0.0198 to 0.0358	0.143	-0.362 to 0.647	1.000
LIM	NC–AD	-0.0080	-0.0419 to 0.0259	-0.142	-0.757 to 0.473	1.000
LIM	MCI–AD	-0.0160	-0.0490 to 0.0170	-0.302	-0.903 to 0.298	1.000
Outdegree hubs	NC–MCI	−0.0030	-0.0074 to 0.0013	-0.412	−0.921 to 0.097	0.49
Outdegree hubs	NC–AD	0.0250	0.0197 to 0.0303	2.753	1.902 to 3.604	<0.001
Outdegree hubs	MCI–AD	0.0281	0.0229 to 0.0332	2.981	2.133 to 3.829	<0.001
Indegree hubs	NC–MCI	−0.0012	−0.0051 to 0.0027	−0.180	−0.685 to 0.325	1.000
Indegree hubs	NC–AD	0.0387	0.0340 to 0.0435	4.950	3.726 to 6.174	<0.001
Indegree hubs	MCI–AD	0.0400	0.0354 to	4.630	3.520 to 5.740	<0.001

Outcome	Comparison	Mean difference	95% CI	Hedges' g	95% CI for g	Bonferroni-corrected *p*-value
			0.0446			

Table S4. Pairwise effect sizes for regional index differences.

ROI	Contrast	Mean difference (95% CI)	p-value	Bonferroni-corrected p-value	Hedges′ g (95% CI)	Hedges′ g 98.33% simultaneous CI
cACG.L	NC-MCI	0.00210 (-0.02257 to 0.02677)	0.866	1.000	0.045 (-0.458 to 0.549)	-0.570 to 0.661
cACG.L	NC-AD	-0.04199 (-0.07208 to -0.01190)	0.007	0.021	-0.837 (-1.477 to -0.197)	-1.618 to -0.055
cACG.L	MCI-AD	-0.04409 (-0.07334 to -0.01484)	0.004	0.011	-0.863 (-1.485 to -0.241)	-1.622 to -0.103
ORBinf.L	NC-MCI	0.00270 (-0.02675 to 0.03215)	0.856	1.000	0.050 (-0.454 to 0.553)	-0.566 to 0.665
ORBinf.L	NC-AD	-0.05393 (-0.08985 to -0.01801)	0.004	0.011	-0.870 (-1.512 to -0.228)	-1.654 to -0.086
ORBinf.L	MCI-AD	-0.05663 (-0.09154 to -0.02172)	0.002	0.006	-0.945 (-1.572 to -0.318)	-1.710 to -0.179
rMFG.L	NC-MCI	0.00370 (-0.02253 to 0.02993)	0.779	1.000	0.071 (-0.432 to 0.575)	-0.544 to 0.687
rMFG.L	NC-AD	-0.07395 (-0.10595 to -0.04195)	<0.001	<0.001	-1.375 (-2.056 to -0.694)	-2.207 to -0.543
rMFG.L	MCI-AD	-0.07765 (-0.10875 to -0.04655)	<0.001	<0.001	-1.533 (-2.206 to -0.860)	-2.355 to -0.711
SMG.L	NC-MCI	0.05189 (0.02816 to 0.07562)	<0.001	<0.001	1.070 (0.531 to 1.609)	0.411 to 1.729
SMG.L	NC-AD	-0.05495 (-0.08389 to -0.02601)	<0.001	<0.001	-1.113 (-1.772 to -0.454)	-1.918 to -0.309
SMG.L	MCI-AD	-0.10684 (-0.13497 to -0.07871)	<0.001	<0.001	-2.487 (-3.268 to -1.707)	-3.441 to -1.534
FP.L	NC-MCI	0.00286 (-0.02063 to 0.02635)	0.809	1.000	0.062 (-0.442 to 0.565)	-0.554 to 0.677
FP.L	NC-AD	-0.05733 (-0.08599 to -0.02867)	<0.001	<0.001	-1.184 (-1.848 to -0.519)	-1.995 to -0.372
FP.L	MCI-AD	-0.06019 (-0.08805 to -0.03233)	<0.001	<0.001	-1.333 (-1.988 to -0.678)	-2.133 to -0.533
cACG.R	NC-MCI	0.00581 (-0.02651 to	0.721	1.000	0.093 (-0.411 to 0.597)	-0.522 to 0.709

ROI	Contrast	Mean difference (95% CI)	p-value	Bonferroni-corrected p-value	Hedges' g (95% CI)	Hedges' g 98.33% simultaneous CI
		0.03813)				
cACG.R	NC-AD	-0.11624 (-0.15566 to -0.07682)	<0.001	<0.001	-1.676 (-2.387 to -0.965)	-2.545 to -0.808
cACG.R	MCI-AD	-0.12205 (-0.16037 to -0.08373)	<0.001	<0.001	-1.984 (-2.703 to -1.264)	-2.862 to -1.105
LOF.R	NC-MCI	-0.01949 (-0.03985 to 0.00087)	0.060	0.181	-0.468 (-0.978 to 0.043)	-1.091 to 0.156
LOF.R	NC-AD	-0.05848 (-0.08332 to -0.03364)	<0.001	<0.001	-1.415 (-2.100 to -0.730)	-2.251 to -0.578
LOF.R	MCI-AD	-0.03899 (-0.06313 to -0.01485)	0.002	0.006	-1.031 (-1.663 to -0.399)	-1.803 to -0.258
LING.R	NC-MCI	0.00204 (-0.02280 to 0.02688)	0.870	1.000	0.042 (-0.462 to 0.546)	-0.573 to 0.657
LING.R	NC-AD	-0.04073 (-0.07103 to -0.01043)	0.009	0.027	-0.893 (-1.537 to -0.250)	-1.679 to -0.108
LING.R	MCI-AD	-0.04277 (-0.07222 to -0.01332)	0.005	0.015	-0.808 (-1.427 to -0.189)	-1.564 to -0.051
PCL.R	NC-MCI	-0.01664 (-0.04289 to 0.00961)	0.210	0.631	-0.323 (-0.830 to 0.184)	-0.942 to 0.296
PCL.R	NC-AD	-0.04992 (-0.08194 to -0.01790)	0.003	0.008	-0.905 (-1.549 to -0.261)	-1.692 to -0.119
PCL.R	MCI-AD	-0.03328 (-0.06440 to -0.00216)	0.036	0.109	-0.666 (-1.279 to -0.054)	-1.414 to 0.081
PoCG.R	NC-MCI	0.04972 (0.01439 to 0.08505)	0.006	0.019	0.682 (0.163 to 1.200)	0.049 to 1.315
PoCG.R	NC-AD	-0.03443 (-0.07752 to 0.00866)	0.116	0.347	-0.503 (-1.127 to 0.120)	-1.265 to 0.258
PoCG.R	MCI-AD	-0.08415 (-0.12604 to -0.04226)	<0.001	<0.001	-1.240 (-1.888 to -0.592)	-2.031 to -0.449
PCG.R	NC-MCI	0.00457 (-0.02985 to 0.03899)	0.792	1.000	0.069 (-0.435 to 0.573)	-0.546 to 0.684
PCG.R	NC-AD	-0.09128 (-0.13326 to -	<0.001	<0.001	-1.247 (-1.916 to -0.577)	-2.065 to -0.429

ROI	Contrast	Mean difference (95% CI)	*p*-value	Bonferroni-corrected *p*-value	Hedges' g (95% CI)	Hedges' g 98.33% simultaneous CI
		0.04930)				
PCG.R	MCI-AD	-0.09585 (-0.13666 to -0.05504)	<0.001	<0.001	-1.451 (-2.116 to -0.785)	-2.263 to -0.638
PreCG.R	NC-MCI	0.04665 (0.02063 to 0.07267)	<0.001	0.002	0.910 (0.380 to 1.440)	0.263 to 1.557
PreCG.R	NC-AD	-0.04939 (-0.08113 to -0.01765)	0.003	0.008	-0.943 (-1.590 to -0.297)	-1.733 to -0.154
PreCG.R	MCI-AD	-0.09604 (-0.12690 to -0.06518)	<0.001	<0.001	-1.868 (-2.574 to -1.161)	-2.730 to -1.005
PCUN.R	NC-MCI	0.05451 (0.02607 to 0.08295)	<0.001	<0.001	0.961 (0.428 to 1.493)	0.310 to 1.611
PCUN.R	NC-AD	0.00287 (-0.03181 to 0.03755)	0.869	1.000	0.047 (-0.567 to 0.661)	-0.704 to 0.797
PCUN.R	MCI-AD	-0.05164 (-0.08536 to -0.01792)	0.003	0.009	-1.008 (-1.639 to -0.377)	-1.779 to -0.238
rMFG.R	NC-MCI	0.00226 (-0.01742 to 0.02194)	0.820	1.000	0.057 (-0.447 to 0.560)	-0.559 to 0.672
rMFG.R	NC-AD	-0.04513 (-0.06914 to -0.02112)	<0.001	0.001	-1.171 (-1.834 to -0.507)	-1.981 to -0.360
rMFG.R	MCI-AD	-0.04739 (-0.07072 to -0.02406)	<0.001	<0.001	-1.234 (-1.881 to -0.587)	-2.024 to -0.443
SFG.R	NC-MCI	0.02559 (0.00705 to 0.04413)	0.007	0.022	0.744 (0.222 to 1.265)	0.107 to 1.380
SFG.R	NC-AD	-0.02709 (-0.04970 to -0.00448)	0.020	0.059	-0.714 (-1.347 to -0.081)	-1.487 to 0.059
SFG.R	MCI-AD	-0.05268 (-0.07466 to -0.03070)	<0.001	<0.001	-1.367 (-2.025 to -0.709)	-2.170 to -0.563
SPG.R	NC-MCI	0.00350 (-0.01942 to 0.02642)	0.762	1.000	0.076 (-0.427 to 0.580)	-0.539 to 0.692
SPG.R	NC-AD	-0.07006 (-0.09802 to -0.04210)	<0.001	<0.001	-1.463 (-2.153 to -0.774)	-2.305 to -0.621
SPG.R	MCI-AD	-0.07356 (-0.10074 to -0.04638)	<0.001	<0.001	-1.724 (-2.415 to -1.033)	-2.568 to -0.880
STG.R	NC-MCI	0.00368 (-	0.785	1.000	0.068 (-0.435 to	-0.547 to 0.684

ROI	Contrast	Mean difference<br><br>(95% CI)	*p*-value	Bonferroni-corrected *p*-value	Hedges′ g<br><br>(95% CI)	Hedges′ g 98.33% simultaneous CI
		0.02307 to 0.03043)			0.572)	
STG.R	NC-AD	-0.07355 (-0.10618 to -0.04092)	<0.001	<0.001	-1.579 (-2.280 to -0.878)	-2.435 to -0.723
STG.R	MCI-AD	-0.07723 (-0.10895 to -0.04551)	<0.001	<0.001	-1.346 (-2.003 to -0.690)	-2.148 to -0.545

Table S5. Correlation between C^e and nodes degree.

ROI	Statistics (*r, p*)	
	**indegree**	**outdegree**
BSTS.L	-0.290,0.010	0.458,<0.001
cACG.L	-0.314,0.005	0.529,<0.001
cMFG.L	-0.423,<0.001	0.463,<0.001
CUN.L	-0.407,<0.001	0.389,<0.001
OLF.L	-0.376,<0.001	0.417,<0.001
FFG.L	-0.310,0.006	0.451,<0.001
IPL.L	-0.370,<0.001	0.533,<0.001
ITG.L	-0.414,<0.001	0.500,<0.001
ICG.L	-0.080,0.490	0.664,<0.001
LOG.L	-0.430,<0.001	0.482,<0.001
LOF.L	-0.550,<0.001	0.406,<0.001
LING.L	-0.475,<0.001	0.517,<0.001
MOF.L	-0.420,<0.001	0.478,<0.001
MTG.L	-0.254,0.026	0.463,<0.001
PHG.L	-0.340,0.002	0.411,<0.001
PCL.L	-0.474,<0.001	0.312,0.006
Poper.L	-0.093,0.422	0.443,<0.001
ORBinf.L	-0.086,0.456	0.582,<0.001
IFGtriang.L	-0.282,0.013	0.394,<0.001
periCAG.L	-0.470,<0.001	0.436,<0.001
PoCG.L	-0.320,0.005	0.595,<0.001
PCG.L	-0.287,0.011	0.464,<0.001
PreCG.L	-0.503,<0.001	0.515,<0.001
PCUN.L	-0.510,<0.001	0.359,0.001
rACG.L	-0.510,<0.001	0.487,<0.001
rMFG.L	-0.540,<0.001	0.293,0.010
SFG.L	-0.672,<0.001	0.334,0.003
SPG.L	-0.495,<0.001	0.474,<0.001
STG.L	-0.493,<0.001	0.543,<0.001
SMG.L	-0.632,<0.001	0.521,<0.001
FP.L	-0.491,<0.001	0.440,<0.001
TP.L	-0.466,<0.001	0.437,<0.001
TTG.L	-0.181,0.116	0.573,<0.001
INS.L	-0.304,0.007	0.668,<0.001
BSTS.R	-0.341,0.002	0.346,0.002
cACG.R	-0.406,<0.001	0.418,<0.001
cMFG.R	-0.381,0.001	0.590, <0.001
CUN.R	-0.287,0.012	0.443, <0.001
OLF.R	-0.311,0.006	0.391, <0.001
FFG.R	-0.462,<0.001	0.490, <0.001
IPL.R	-0.365,0.001	0.535, <0.001
ITG.R	-0.428,<0.001	0.437, <0.001
ICG.R	-0.525,<0.001	0.427, <0.001
LOG.R	-0.355,0.002	0.476, <0.001
LOF.R	-0.345,0.002	0.486, <0.001
LING.R	-0.485,<0.001	0.454, <0.001
MOF.R	-0.268,0.018	0.316, 0.005
MTG.R	-0.518,<0.001	0.577, <0.001
PHG.R	-0.247,0.030	0.473, <0.001
PCL.R	-0.523,<0.001	0.441, <0.001

ROI	Statistics (*r, p*)	
	**indegree**	**outdegree**
Poper.R	-0.444,<0.001	0.504, <0.001
ORBinf.R	-0.409,<0.001	0.523,<0.001
IFGtriang.R	-0.418,<0.001	0.382,<0.001
periCAG.R	-0.347,0.002	0.539,<0.001
PoCG.R	-0.231,0.043	0.536,<0.001
PCG.R	-0.223,0.051	0.427,<0.001
PreCG.R	-0.334,0.003	0.572,<0.001
PCUN.R	-0.549,<0.001	0.360,0.001
rACG.R	-0.392,<0.001	0.449,<0.001
rMFG.R	-0.475,<0.001	0.448,<0.001
SFG.R	-0.551,<0.001	0.478,<0.001
SPG.R	-0.512,<0.001	0.357,0.001
STG.R	-0.445,<0.001	0.431,<0.001
SMG.R	-0.393,<0.001	0.474,<0.001
FP.R	-0.367,0.001	0.442,<0.001
TP.R	-0.397,<0.001	0.360,0.001
TTG.R	-0.541,<0.001	0.132,0.251
INS.R	-0.624,<0.001	0.322,0.004

Table S6. Complete hierarchical second-order polynomial regression results.

ROI	Clinical score	Covariate-model R^2	Full-model R^2	ΔR^2	F-change (2, 72)	p	BH-adjusted p
Whole brain	CDR	0.055	0.113	0.058	2.354	0.1023	0.1023
Whole brain	FAQ	0.070	0.141	0.071	2.976	0.0573	0.0860
Whole brain	MMSE	0.085	0.191	0.106	4.717	0.0119	0.0357
BSTS.L	CDR	0.055	0.064	0.009	0.335	0.7167	0.7202
BSTS.L	FAQ	0.070	0.080	0.010	0.387	0.6804	0.6940
BSTS.L	MMSE	0.085	0.101	0.016	0.637	0.5320	0.5811
cACG.L	CDR	0.055	0.098	0.043	1.716	0.1870	0.3909
cACG.L	FAQ	0.070	0.103	0.033	1.324	0.2724	0.4184
cACG.L	MMSE	0.085	0.179	0.094	4.122	0.0202	0.1872
cMFG.L	CDR	0.055	0.079	0.024	0.922	0.4024	0.4945
cMFG.L	FAQ	0.070	0.109	0.039	1.559	0.2173	0.3927
cMFG.L	MMSE	0.085	0.124	0.039	1.609	0.2073	0.3909
CUN.L	CDR	0.055	0.096	0.041	1.613	0.2063	0.3909
CUN.L	FAQ	0.070	0.123	0.053	2.162	0.1225	0.3471
CUN.L	MMSE	0.085	0.138	0.053	2.208	0.1173	0.3402
OLF.L	CDR	0.055	0.077	0.022	0.845	0.4336	0.5143
OLF.L	FAQ	0.070	0.110	0.040	1.601	0.2087	0.3909
OLF.L	MMSE	0.085	0.124	0.039	1.613	0.2063	0.3909
FFG.L	CDR	0.055	0.071	0.016	0.620	0.5409	0.5811
FFG.L	FAQ	0.070	0.098	0.028	1.116	0.3333	0.4387
FFG.L	MMSE	0.085	0.111	0.026	1.054	0.3538	0.4562
IPL.L	CDR	0.055	0.073	0.018	0.717	0.4917	0.5514
IPL.L	FAQ	0.070	0.099	0.029	1.177	0.3141	0.4371
IPL.L	MMSE	0.085	0.103	0.018	0.716	0.4920	0.5514
ITG.L	CDR	0.055	0.080	0.025	0.984	0.3787	0.4739
ITG.L	FAQ	0.070	0.112	0.042	1.682	0.1933	0.3909
ITG.L	MMSE	0.085	0.129	0.044	1.839	0.1664	0.3888
ICG.L	CDR	0.055	0.076	0.021	0.805	0.4509	0.5287
ICG.L	FAQ	0.070	0.086	0.016	0.610	0.5459	0.5811
ICG.L	MMSE	0.085	0.102	0.017	0.698	0.5010	0.5585
LOG.L	CDR	0.055	0.068	0.013	0.487	0.6167	0.6458
LOG.L	FAQ	0.070	0.087	0.017	0.689	0.5053	0.5599
LOG.L	MMSE	0.085	0.106	0.021	0.860	0.4274	0.5099
LOF.L	CDR	0.055	0.122	0.067	2.741	0.0712	0.3136
LOF.L	FAQ	0.070	0.110	0.040	1.629	0.2032	0.3909
LOF.L	MMSE	0.085	0.104	0.019	0.772	0.4657	0.5429
LING.L	CDR	0.055	0.108	0.053	2.134	0.1258	0.3489
LING.L	FAQ	0.070	0.118	0.048	1.942	0.1509	0.3674
LING.L	MMSE	0.085	0.133	0.048	1.977	0.1460	0.3674
MOF.L	CDR	0.055	0.071	0.016	0.629	0.5361	0.5811
MOF.L	FAQ	0.070	0.108	0.038	1.529	0.2238	0.3927

ROI	Clinical score	Covariate-model R^2	Full-model R^2	ΔR^2	F-change (2, 72)	*p*	BH-adjusted *p*
MOF.L	MMSE	0.085	0.132	0.047	1.942	0.1508	0.3674
MTG.L	CDR	0.055	0.083	0.028	1.118	0.3325	0.4387
MTG.L	FAQ	0.070	0.112	0.042	1.721	0.1861	0.3909
MTG.L	MMSE	0.085	0.133	0.048	1.978	0.1458	0.3674
PHG.L	CDR	0.055	0.074	0.019	0.733	0.4839	0.5514
PHG.L	FAQ	0.070	0.107	0.037	1.479	0.2346	0.3968
PHG.L	MMSE	0.085	0.134	0.049	2.049	0.1363	0.3610
PCL.L	CDR	0.055	0.124	0.069	2.844	0.0647	0.3136
PCL.L	FAQ	0.070	0.125	0.055	2.278	0.1099	0.3296
PCL.L	MMSE	0.085	0.118	0.033	1.357	0.2640	0.4143
pOPER.L	CDR	0.055	0.057	0.002	0.088	0.9156	0.9156
pOPER.L	FAQ	0.070	0.098	0.028	1.130	0.3288	0.4387
pOPER.L	MMSE	0.085	0.107	0.022	0.896	0.4126	0.4996
ORBinf.L	CDR	0.055	0.086	0.031	1.221	0.3010	0.4294
ORBinf.L	FAQ	0.070	0.127	0.057	2.351	0.1026	0.3270
ORBinf.L	MMSE	0.085	0.144	0.059	2.481	0.0908	0.3138
IFGtriang.L	CDR	0.055	0.071	0.016	0.622	0.5399	0.5811
IFGtriang.L	FAQ	0.070	0.108	0.038	1.522	0.2252	0.3927
IFGtriang.L	MMSE	0.085	0.120	0.035	1.412	0.2503	0.4020
periCAG.L	CDR	0.055	0.067	0.012	0.477	0.6225	0.6458
periCAG.L	FAQ	0.070	0.082	0.012	0.476	0.6230	0.6458
periCAG.L	MMSE	0.085	0.094	0.009	0.356	0.7016	0.7086
PoCG.L	CDR	0.055	0.157	0.102	4.356	0.0164	0.1872
PoCG.L	FAQ	0.070	0.106	0.036	1.429	0.2463	0.4020
PoCG.L	MMSE	0.085	0.128	0.043	1.779	0.1761	0.3909
PCG.L	CDR	0.055	0.078	0.023	0.889	0.4155	0.4996
PCG.L	FAQ	0.070	0.136	0.066	2.754	0.0704	0.3136
PCG.L	MMSE	0.085	0.114	0.029	1.197	0.3080	0.4363
PreCG.L	CDR	0.055	0.082	0.027	1.049	0.3556	0.4562
PreCG.L	FAQ	0.070	0.117	0.047	1.937	0.1515	0.3674
PreCG.L	MMSE	0.085	0.147	0.062	2.638	0.0784	0.3136
PCUN.L	CDR	0.055	0.087	0.032	1.259	0.2900	0.4196
PCUN.L	FAQ	0.070	0.106	0.036	1.465	0.2379	0.3968
PCUN.L	MMSE	0.085	0.151	0.066	2.814	0.0666	0.3136
rACG.L	CDR	0.055	0.094	0.039	1.556	0.2180	0.3927
rACG.L	FAQ	0.070	0.124	0.054	2.208	0.1173	0.3402
rACG.L	MMSE	0.085	0.146	0.061	2.570	0.0835	0.3138
rMFG.L	CDR	0.055	0.087	0.032	1.262	0.2893	0.4196
rMFG.L	FAQ	0.070	0.149	0.079	3.342	0.0409	0.2794
rMFG.L	MMSE	0.085	0.261	0.176	8.574	0.0005	0.0311
SFG.L	CDR	0.055	0.107	0.052	2.103	0.1295	0.3523
SFG.L	FAQ	0.070	0.132	0.062	2.555	0.0847	0.3138

ROI	Clinical score	Covariate-model R^2	Full-model R^2	ΔR^2	F-change (2, 72)	*p*	BH-adjusted *p*
SFG.L	MMSE	0.085	0.156	0.071	3.038	0.0541	0.3136
SPG.L	CDR	0.055	0.103	0.048	1.927	0.1531	0.3674
SPG.L	FAQ	0.070	0.166	0.096	4.133	0.0200	0.1872
SPG.L	MMSE	0.085	0.107	0.022	0.897	0.4125	0.4996
STG.L	CDR	0.055	0.095	0.040	1.606	0.2078	0.3909
STG.L	FAQ	0.070	0.087	0.017	0.684	0.5078	0.5599
STG.L	MMSE	0.085	0.132	0.047	1.928	0.1529	0.3674
SMG.L	CDR	0.055	0.179	0.124	5.437	0.0063	0.1128
SMG.L	FAQ	0.070	0.268	0.198	9.738	0.0002	0.0311
SMG.L	MMSE	0.085	0.229	0.144	6.724	0.0021	0.0562
FP.L	CDR	0.055	0.097	0.042	1.674	0.1946	0.3909
FP.L	FAQ	0.070	0.113	0.043	1.745	0.1819	0.3909
FP.L	MMSE	0.085	0.148	0.063	2.662	0.0767	0.3136
TP.L	CDR	0.055	0.083	0.028	1.118	0.3327	0.4387
TP.L	FAQ	0.070	0.100	0.030	1.188	0.3109	0.4371
TP.L	MMSE	0.085	0.118	0.033	1.330	0.2708	0.4184
TTG.L	CDR	0.055	0.116	0.061	2.504	0.0889	0.3138
TTG.L	FAQ	0.070	0.148	0.078	3.302	0.0425	0.2794
TTG.L	MMSE	0.085	0.151	0.066	2.807	0.0670	0.3136
INS.L	CDR	0.055	0.095	0.040	1.603	0.2085	0.3909
INS.L	FAQ	0.070	0.134	0.064	2.682	0.0753	0.3136
INS.L	MMSE	0.085	0.140	0.055	2.298	0.1078	0.3296
BSTS.R	CDR	0.055	0.083	0.028	1.117	0.3329	0.4387
BSTS.R	FAQ	0.070	0.108	0.038	1.525	0.2246	0.3927
BSTS.R	MMSE	0.085	0.130	0.045	1.863	0.1627	0.3859
cACG.R	CDR	0.055	0.122	0.067	2.747	0.0708	0.3136
cACG.R	FAQ	0.070	0.250	0.180	8.640	0.0004	0.0311
cACG.R	MMSE	0.085	0.228	0.143	6.668	0.0022	0.0562
cMFG.R	CDR	0.055	0.078	0.023	0.887	0.4164	0.4996
cMFG.R	FAQ	0.070	0.121	0.051	2.079	0.1325	0.3557
cMFG.R	MMSE	0.085	0.143	0.058	2.441	0.0943	0.3145
CUN.R	CDR	0.055	0.067	0.012	0.475	0.6236	0.6458
CUN.R	FAQ	0.070	0.079	0.009	0.365	0.6957	0.7061
CUN.R	MMSE	0.085	0.100	0.015	0.609	0.5469	0.5811
OLF.R	CDR	0.055	0.070	0.015	0.598	0.5525	0.5840
OLF.R	FAQ	0.070	0.106	0.036	1.459	0.2393	0.3968
OLF.R	MMSE	0.085	0.120	0.035	1.414	0.2498	0.4020
FFG.R	CDR	0.055	0.073	0.018	0.718	0.4911	0.5514
FFG.R	FAQ	0.070	0.088	0.018	0.719	0.4908	0.5514
FFG.R	MMSE	0.085	0.121	0.036	1.462	0.2387	0.3968
IPL.R	CDR	0.055	0.087	0.032	1.280	0.2842	0.4184
IPL.R	FAQ	0.070	0.108	0.038	1.533	0.2228	0.3927

ROI	Clinical score	Covariate-model R^2	Full-model R^2	ΔR^2	F-change (2, 72)	*p*	BH-adjusted *p*
IPL.R	MMSE	0.085	0.128	0.043	1.770	0.1776	0.3909
ITG.R	CDR	0.055	0.079	0.024	0.931	0.3987	0.4929
ITG.R	FAQ	0.070	0.107	0.037	1.506	0.2286	0.3953
ITG.R	MMSE	0.085	0.119	0.034	1.403	0.2525	0.4024
ICG.R	CDR	0.055	0.067	0.012	0.456	0.6354	0.6546
ICG.R	FAQ	0.070	0.086	0.016	0.612	0.5453	0.5811
ICG.R	MMSE	0.085	0.106	0.021	0.828	0.4410	0.5200
LOG.R	CDR	0.055	0.085	0.030	1.160	0.3192	0.4371
LOG.R	FAQ	0.070	0.089	0.019	0.735	0.4831	0.5514
LOG.R	MMSE	0.085	0.108	0.023	0.948	0.3924	0.4881
LOF.R	CDR	0.055	0.183	0.128	5.640	0.0053	0.1082
LOF.R	FAQ	0.070	0.136	0.066	2.750	0.0706	0.3136
LOF.R	MMSE	0.085	0.111	0.026	1.053	0.3542	0.4562
LING.R	CDR	0.055	0.145	0.090	3.789	0.0272	0.2190
LING.R	FAQ	0.070	0.161	0.091	3.905	0.0245	0.2087
LING.R	MMSE	0.085	0.199	0.114	5.124	0.0083	0.1304
MOF.R	CDR	0.055	0.085	0.030	1.168	0.3167	0.4371
MOF.R	FAQ	0.070	0.126	0.056	2.304	0.1072	0.3296
MOF.R	MMSE	0.085	0.148	0.063	2.678	0.0755	0.3136
MTG.R	CDR	0.055	0.087	0.032	1.282	0.2837	0.4184
MTG.R	FAQ	0.070	0.113	0.043	1.748	0.1814	0.3909
MTG.R	MMSE	0.085	0.155	0.070	3.000	0.0560	0.3136
PHG.R	CDR	0.055	0.087	0.032	1.281	0.2839	0.4184
PHG.R	FAQ	0.070	0.124	0.054	2.198	0.1184	0.3402
PHG.R	MMSE	0.085	0.140	0.055	2.292	0.1084	0.3296
PCL.R	CDR	0.055	0.198	0.143	6.419	0.0027	0.0617
PCL.R	FAQ	0.070	0.180	0.110	4.829	0.0108	0.1568
PCL.R	MMSE	0.085	0.155	0.070	2.982	0.0570	0.3136
pOPER.R	CDR	0.055	0.084	0.029	1.136	0.3267	0.4387
pOPER.R	FAQ	0.070	0.130	0.060	2.491	0.0900	0.3138
pOPER.R	MMSE	0.085	0.145	0.060	2.511	0.0883	0.3138
ORBinf.R	CDR	0.055	0.085	0.030	1.163	0.3183	0.4371
ORBinf.R	FAQ	0.070	0.113	0.043	1.738	0.1831	0.3909
ORBinf.R	MMSE	0.085	0.117	0.032	1.289	0.2819	0.4184
IFGtriang.R	CDR	0.055	0.066	0.011	0.428	0.6537	0.6701
IFGtriang.R	FAQ	0.070	0.095	0.025	0.993	0.3756	0.4730
IFGtriang.R	MMSE	0.085	0.104	0.019	0.752	0.4749	0.5505
periCAG.R	CDR	0.055	0.086	0.031	1.222	0.3007	0.4294
periCAG.R	FAQ	0.070	0.110	0.040	1.625	0.2041	0.3909
periCAG.R	MMSE	0.085	0.136	0.051	2.128	0.1266	0.3489
PoCG.R	CDR	0.055	0.217	0.162	7.448	0.0011	0.0524
PoCG.R	FAQ	0.070	0.131	0.061	2.527	0.0870	0.3138

ROI	Clinical score	Covariate-model R^2	Full-model R^2	ΔR^2	F-change (2, 72)	*p*	BH-adjusted *p*
PoCG.R	MMSE	0.085	0.169	0.084	3.639	0.0312	0.2359
PCG.R	CDR	0.055	0.095	0.040	1.591	0.2108	0.3909
PCG.R	FAQ	0.070	0.170	0.100	4.337	0.0167	0.1872
PCG.R	MMSE	0.085	0.124	0.039	1.603	0.2084	0.3909
PreCG.R	CDR	0.055	0.116	0.061	2.484	0.0905	0.3138
PreCG.R	FAQ	0.070	0.158	0.088	3.762	0.0279	0.2190
PreCG.R	MMSE	0.085	0.184	0.099	4.368	0.0162	0.1872
PCUN.R	CDR	0.055	0.100	0.045	1.800	0.1727	0.3909
PCUN.R	FAQ	0.070	0.118	0.048	1.959	0.1484	0.3674
PCUN.R	MMSE	0.085	0.179	0.094	4.122	0.0202	0.1872
rACG.R	CDR	0.055	0.092	0.037	1.480	0.2344	0.3968
rACG.R	FAQ	0.070	0.128	0.058	2.411	0.0970	0.3145
rACG.R	MMSE	0.085	0.161	0.076	3.267	0.0439	0.2796
rMFG.R	CDR	0.055	0.127	0.072	2.969	0.0577	0.3136
rMFG.R	FAQ	0.070	0.217	0.147	6.759	0.0020	0.0562
rMFG.R	MMSE	0.085	0.160	0.075	3.214	0.0460	0.2845
SFG.R	CDR	0.055	0.148	0.093	3.930	0.0240	0.2087
SFG.R	FAQ	0.070	0.166	0.096	4.144	0.0198	0.1872
SFG.R	MMSE	0.085	0.204	0.119	5.382	0.0066	0.1128
SPG.R	CDR	0.055	0.139	0.084	3.512	0.0350	0.2553
SPG.R	FAQ	0.070	0.227	0.157	7.312	0.0013	0.0524
SPG.R	MMSE	0.085	0.129	0.044	1.819	0.1696	0.3888
STG.R	CDR	0.055	0.124	0.069	2.836	0.0653	0.3136
STG.R	FAQ	0.070	0.115	0.045	1.831	0.1677	0.3888
STG.R	MMSE	0.085	0.154	0.069	2.936	0.0595	0.3136
SMG.R	CDR	0.055	0.114	0.059	2.409	0.0971	0.3145
SMG.R	FAQ	0.070	0.174	0.104	4.541	0.0139	0.1872
SMG.R	MMSE	0.085	0.162	0.077	3.306	0.0423	0.2794
FP.R	CDR	0.055	0.081	0.026	1.015	0.3676	0.4687
FP.R	FAQ	0.070	0.102	0.032	1.302	0.2784	0.4184
FP.R	MMSE	0.085	0.116	0.031	1.277	0.2851	0.4184
TP.R	CDR	0.055	0.092	0.037	1.447	0.2420	0.3981
TP.R	FAQ	0.070	0.109	0.039	1.595	0.2100	0.3909
TP.R	MMSE	0.085	0.123	0.038	1.576	0.2138	0.3927
TTG.R	CDR	0.055	0.081	0.026	1.007	0.3704	0.4694
TTG.R	FAQ	0.070	0.102	0.032	1.297	0.2797	0.4184
TTG.R	MMSE	0.085	0.118	0.033	1.358	0.2637	0.4143
INS.R	CDR	0.055	0.082	0.027	1.054	0.3540	0.4562
INS.R	FAQ	0.070	0.134	0.064	2.642	0.0781	0.3136
INS.R	MMSE	0.085	0.144	0.059	2.462	0.0924	0.3142

Table S7A. Whole-brain C across the six-setting local sensitivity window.

Fusion setting	NC mean±SD	MCI mean±SD	AD mean±SD	F	Overall P	NC–MCI Padj	NC–AD Padj	MCI–AD Padj
α=0.7, β=0.1	0.98993±0.00450	0.98858±0.00456	0.99345±0.00451	6.26	0.003	0.748	0.046	0.002
α=0.8, β=0.1	0.99000±0.00470	0.98870±0.00460	0.99360±0.00440	6.16	0.003	0.824	0.044	0.002
α=0.9, β=0.1	0.99011±0.00454	0.98882±0.00452	0.99345±0.00429	5.78	0.005	0.798	0.060	0.003
α=0.7, β=0.2	0.99023±0.00466	0.98925±0.00473	0.99332±0.00447	4.17	0.019	1.000	0.112	0.016
α=0.8, β=0.2	0.99025±0.00458	0.98890±0.00447	0.99352±0.00433	5.73	0.005	0.735	0.068	0.003
α=0.9, β=0.2	0.99035±0.00474	0.98938±0.00468	0.99332±0.00444	3.90	0.025	1.000	0.136	0.021

Table S7B. Whole-brain C across five exact nominal threshold anchors.

Exact threshold	NC mean±SD	MCI mean±SD	AD mean±SD	F	Overall *P*	NC–MCI Padj	NC–AD Padj	MCI–AD Padj
10%	0.98930±0.00474	0.98806±0.00460	0.99290±0.00455	5.91	0.004	0.905	0.047	0.003
16%	0.98971±0.00466	0.98845±0.00456	0.99328±0.00444	6.06	0.004	0.861	0.045	0.003
22%	0.99000±0.00470	0.98870±0.00460	0.99360±0.00440	6.16	0.003	0.824	0.044	0.002
28%	0.99033±0.00462	0.98937±0.00473	0.99390±0.00436	5.30	0.007	1.000	0.048	0.006
35%	0.99063±0.00454	0.98970±0.00464	0.99408±0.00429	5.13	0.008	1.000	0.053	0.007

Table S8. AD threshold sensitivity at five exact nominal anchors.

Exact threshold	Minimum AD subjects	Edges	Jaccard	Dice	Bootstrap Jaccard [95% CI]
10%	2	34	0.618	0.764	0.58 [0.41, 0.69]
16%	3	27	0.778	0.875	0.67 [0.53, 0.77]
22%	4	21	1.000	1.000	0.72 [0.60, 0.81]
28%	5	17	0.810	0.895	0.75 [0.62, 0.84]
35%	6	11	0.524	0.688	0.61 [0.44, 0.72]

Table S9. Cross-threshold prevalence of the reported driving nodes.

Group	ROI	RSN	Exact threshold anchors present, n/5
NC	BSTS.L	DMN	4
NC	SFG.L	DMN	4
NC	MTG.R	DMN	5
NC	cACG.R	VAN	3
NC	INS.R	VAN	4
NC	LING.R	VN	3
NC	TP.L	LIM	3
NC	LOF.L	LIM	3
MCI	IPL.R	DMN	4
MCI	MTG.R	DMN	5
MCI	PCUN.R	DMN	4
MCI	PreCG.L	SMN	4
MCI	TTG.L	SMN	3
MCI	PoCG.R	SMN	3
MCI	INS.L	VAN	4
MCI	cACG.R	VAN	3
MCI	SMG.L	VAN	3
AD	IPL.R	DMN	3
AD	MTG.R	DMN	5
AD	STG.L	SMN	3
AD	SMG.R	VAN	3
AD	cMFG.R	FPN	4

Table S10. Descriptive associations of *C* with matching-derived measures.

Comparison	Model	Statistic	Coefficient	*p* value
Driver-node fraction	Pooled, unadjusted	Spearman ϱ	−0.579	<0.001
Driver-node fraction	Adjusted for diagnosis and network density	Partial rank r	−0.561	<0.001
Maximum-matching ratio	Pooled, unadjusted	Spearman ϱ	0.209	0.068
Maximum-matching ratio	Adjusted for diagnosis and network density	Partial rank r	0.031	0.786
